# Group dance interventions for community dwelling older adults to prevent and treat sarcopenia: a mixed methods systematic review

**DOI:** 10.1007/s11357-025-02072-z

**Published:** 2026-01-13

**Authors:** Nan Hua, Ruth Harris, Shalini Ahuja, Xiangmin Tan, Kia-Chong Chua, Yihan Mo, Joanne M. Fitzpatrick

**Affiliations:** 1 Care for Long Term Conditions Research Division, Florence Nightingale Faculty of Nursing, Midwifery & Palliative Care, King’s College London, London, UK; 2 Methodologies Research Division, Florence Nightingale Faculty of Nursing, Midwifery & Palliative Care, King’s College London, London, UK; 3 School of Rural Health, Monash University, Warragul, VIC Australia; 4 Biostatistics & Health Informatics, Institute of Psychiatry, Psychology & Neuroscience, King’s College London, London, UK; 5 Cicely Saunders Institute of Palliative Care, Policy and Rehabilitation , Florence Nightingale Faculty of Nursing, Midwifery and Palliative Care, King’s College London, London, UK

**Keywords:** Group dance, Sarcopenia, Community intervention, Older adults, Systematic review, Mixed methods

## Abstract

Group dance is an engaging physical activity that may benefit the prevention and management of sarcopenia among older adults. However, current evidence on this topic is limited. We aimed to evaluate published evidence on group dance for sarcopenia in community dwelling older adults. Studies published in English and Chinese between 2014 and 2024 were identified through searches in MEDLINE, Embase, and PsycINFO via Ovid, CINAHL via EBSCO, Chinese National Knowledge Infrastructure, Wan Fang, Google Scholar, and Web of Science Core Collection. Two reviewers independently performed screening, data extraction, and risk of bias assessment. Quantitative data were meta-analysed, and qualitative findings were analysed using thematic analysis. The protocol was registered with PROSPERO (CRD42024554152). 24 studies were included, comprising 1840 participants across 12 countries and involving nine types of group dance interventions. Meta-analysis showed significant improvements in the Short Physical Performance Battery (MD = 1.32, 95%CI: 0.56, 2.08, I^2^ = 37%), Sit and Reach Test (MD = 1.91, 95%CI: 0.29, 2.91, I^2^ = 0), Montreal Cognitive Assessment (MD = 0.94, 95%CI: 0.01, 1.87, I^2^ = 0) and Trail Making Test (SMD = 0.12, 95%CI: 0.03, 0.21, I^2^ = 34%). No significant differences were observed in muscle strength, 5 times Sit-To-Stand, Time Up and Go, Single Leg Test, 30s Chair Stand, and gait speed. Qualitative analysis identified three overarching themes: psychosocial, physical, and cognitive wellbeing. Group dance is a safe, acceptable, and promising community intervention that improves physical performance in older adults. Enjoyment-driven and socially engaging group dance interventions are encouraged to support the prevention and management of sarcopenia. Future investigation should use sarcopenia-specific assessments, and consider cultural context, baseline physical status, and individual preferences when designing and evaluating group dance interventions.

**Graphical Abstract:**

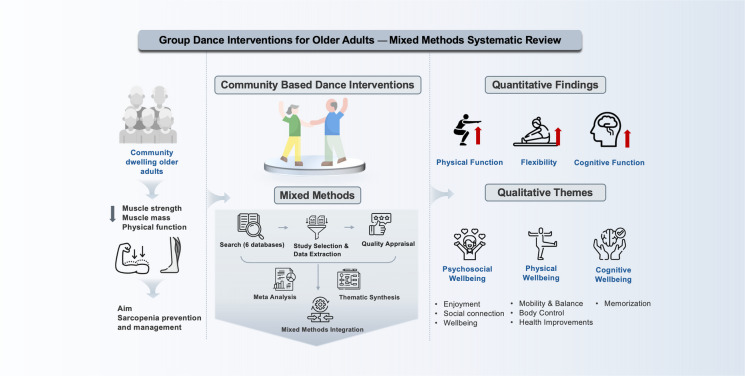

**Supplementary Information:**

The online version contains supplementary material available at 10.1007/s11357-025-02072-z.

## Introduction

Ageing well is a globally relevant public health and socio-economic issue [1]. Key to this is developing and maintaining functional ability, however, this can be compromised by sarcopenia, the age-related loss of skeletal muscle mass and strength, and/or poor physical performance. Sarcopenia poses a significant public health challenge [2] and increases the risk of falls, fractures, physical disability [3, 4], and reduced quality of life (QoL) [5]. The global prevalence of sarcopenia in community-dwelling older adults is estimated to be 10% [6]. The prevention and treatment of sarcopenia is therefore paramount, particularly considering that by 2050, the world population aged 60 years and over is projected to be up to 22% of the entire population [7].

Physical activity is an effective non-pharmacological intervention in the prevention and treatment of sarcopenia [8, 9]. Studies have shown that various forms of exercise, such as resistance training, aerobic exercise, and whole-body vibration training, have potential benefits for improving muscle strength, muscle mass, and balance, as well as preventing or delaying the development of sarcopenia [10, 11]. However, the actual effectiveness of these interventions varies significantly influenced by participants’ health status, exercise regimens, and adherence to interventions [12]. Some exercises (e.g., resistance band training, bodyweight training) often require specialized equipment, which may limit their implementation in community settings. Moreover, a lack of enjoyment and group connection often leads to low participation and completion rates [13], further reducing the health benefits. Therefore, an effective, easily accessible, and engaging intervention to prevent and treat sarcopenia in older adults is imperative.

Innovative activities for older adults such as dance, tai chi, and yoga have been attracting increasing attention. These activities improve both physical function and the overall quality of life for older adults by encouraging psychological and emotional interaction [14–16]. Compared to single-mode exercises, group dance is more engaging and interactive [17]. It may improve older adults’ exercise adherence and social connection, leading to potential long-term benefits. Group dance is a moderate-intensity aerobic exercise. It combines music, rhythm, and social interaction, making it particularly suitable for older adults living in community settings. Studies have shown that regular participation in group dance helps to enhance lower limb muscle strength [18], balance [19], and mental wellbeing [14]. Group dance interventions have also shown notable benefits in managing chronic conditions (e.g., cardiovascular disease [20], Alzheimer’s disease [21]) and enhancing quality of life in older adults, showing its dual role in physical and emotional support [14].

However, current research on the role of group dance in the prevention and management of sarcopenia is limited. Some research [22, 23] has focused on single exercises like resistance or aerobic training, overlooking the social and emotional benefits of group-based activities. Additionally, some reviews [24, 25] have demonstrated the positive effects of dance on physical function, but evidence specific to sarcopenia and comparisons across studies are lacking. It is not currently known if group dance intervention is effective in preventing and treating sarcopenia for older adults, and how it is regarded by this population. We have not identified a systematic review that addresses this important topic. The aim of this original and innovative mixed methods systematic review is to evaluate the evidence on group dance for sarcopenia in community dwelling older adults. Addressing this evidence gap will inform the development and feasibility testing of a novel intervention on this topic. The objectives of this review are to explore the effectiveness of group dance interventions on sarcopenia-related outcomes, such as muscle strength, muscle mass, and physical performance, and to investigate participants’ views, experiences, and adherence to understand the feasibility and acceptability of dance interventions.

## Methods

### Methodological framework and protocol

This review was conducted using the University of York Centre for Reviews and Dissemination [26] and reported in accordance with the Preferred Reporting Items for Systematic Review and Meta-Analysis (PRISMA) [27]. The protocol is registered in PROSPERO (CRD42024554152) [28].

### Inclusion and exclusion criteria

We included studies that investigated group dance interventions delivered in real world community settings for older adults (aged ≥ 60 years) who were community dwelling (living in their own home or with family members). Eligible participants were either diagnosed with sarcopenia with no restrictions on measures used or at risk of sarcopenia as age is the most important risk factor [29]). Studies were included only if participants were physically able to participate in dance activities and had the cognitive ability to understand instructions, confirmed through medical records or clinical assessments conducted by healthcare professionals. There were no restrictions for comparators e.g. other forms of exercise such as walking and exergame, control groups like waiting list control (participants assigned to a waitlist for intervention) or no intervention, and other supportive interventions (e.g., usual care, health education). Studies were required to report at least one sarcopenia specific outcome (e.g., muscle mass, muscle strength, physical performance). All study designs meeting the eligibility criteria were included, including quantitative, qualitative, and mixed-methods studies.

Exclusion criteria were studies involving older adults living in long-term care facilities (e.g., care homes), as well as studies that included participants with serious comorbidities (e.g. cardiovascular and respiratory conditions) restricting physical activity or with clinically diagnosed mental health conditions. Interventions that did not include a structured dance component (e.g., music-assisted exergame-based exercises) were excluded. Editorials, commentaries, opinion pieces, protocols, conference abstracts, quality improvement reports, and systematic reviews were also excluded.

### Information sources

Given resource limitations (e.g., translation costs, time constraints), this study focused on English and Chinese databases. Additionally, as this review represents the initial phase of a doctoral project conducted by the first author (NH), and the subsequent intervention will be developed and operationalised in China, including Chinese literature was essential to ensure adequate representation of evidence relevant to the Chinese context.

For electronic English language databases, we searched MEDLINE, Embase and PsycINFO via Ovid, and CINAHL (nursing and allied health professions) via EBSCO. The Chinese National Knowledge Infrastructure (CNKI) and Wan Fang Database as the widely used databases in China, were also searched to capture relevant studies published in Chinese. Grey literature was searched using Google Scholar and Web of Science Core Collection.

### Search strategy

Searches were conducted for the period 2014–2024 to ensure that the included studies were based on contemporary and comparable definitions of sarcopenia. During this period, the European Working Group on Sarcopenia in Older People updated its diagnostic criteria [2], while the Asian Working Group for Sarcopenia established Asia-specific criteria in 2014 [30] and subsequently refined them in 2019 [29], aiming to improve consistency in how sarcopenia is defined and assessed. Studies published prior to 2014 often used outdated or heterogeneous criteria, which may contribute to variations in reported outcomes. Further, research on exercise and dance-based interventions has expanded considerably during this period. The final search was conducted on 26 June 2024. Snowballing was used to identify relevant sources from included studies. The search strategy was developed and finalised in collaboration with a university library specialist, drawing on PICO concepts to structure the key search terms. A combination of keywords and Medical Subject Headings (MeSH) terms were used, including: “sarcopenia”, “older adults”, “aged”, “dancing”, “management”, “prevention”, and “treatment”. Boolean operators (AND/OR) were applied to combine terms, and search strings were adapted for each database. Detailed search strategies for all databases are presented in Supplementary Materials A.

### Study selection and data extraction

All results were imported into Covidence (www.covidence.org) after duplicate records had been removed using EndNote. Using the eligibility criteria two reviewers independently screened the titles and abstracts (NH, YM) followed by full text review of potentially relevant studies (NH, XT). Any disagreements or uncertainties between screeners were discussed and resolved through consultation with research team members (JF, RH, SA).

A bespoke data extraction tool was piloted independently by two reviewers (NH, XT) using two studies, and duplicate items were removed. All data were extracted by one reviewer (NH) and 20% (n = 5) of studies were independently checked by a second reviewer (XT).

Data extracted were: 1) author(s), publication year, country; 2) study characteristics (aim, objectives, design, inclusion and exclusion criteria, recruitment procedures, allocation); 3) participant characteristics (sample size, age, gender, health status); 4) target outcomes: primary outcomes (muscle mass, muscle strength, physical performance), secondary outcomes (psychological outcomes, social outcomes, body composition, quality of life (QoL), adverse events, economic outcomes), and assessment tools. Intervention characteristics were extracted using the TIDieR Checklist [31], including dance name, rationale for the intervention, what materials were used, dance procedures, and who provided, how the intervention was delivered, where, when and how much, any tailoring or modification during implementation, and fidelity (how well planned, and how well actual). Views and experiences of the dance interventions, reported as themes and subthemes, were also extracted.

### Risk of bias assessment

Risk of bias was assessed by two independent reviewers (NH and XT), with each responsible as primary assessor for 13 records and second assessor for 11 records. The Critical Appraisal Skills Programme (CASP) checklists [32] were used to evaluate Randomized Controlled Trial studies (RCTs) and qualitative studies. The Risk of bias In Non-randomized Studies of Interventions (ROBINS-I) tool was employed to evaluate quasi-experimental studies. For mixed methods studies, the CASP checklist for qualitative studies was used as only the qualitative component met the inclusion criteria for this review. The final evaluation results were reviewed and then confirmed by three supervising reviewers (JF, RH, and SA).

### Analysis and synthesis

To synthesize findings from included studies to evaluate group dance interventions, Review Manager (RevMan5.4) was used to analyse the extracted quantitative data. The meta-analysis was conducted when at least two studies assessed the same outcome, following the guidelines of the Cochrane handbook for systematic reviews of interventions for model selection, subgroup analysis, sensitivity analysis, and results interpretation [33]. Data that could be statistically combined were continuous variables. Mean differences (MDs) with 95% confidence intervals (CIs) were used to calculate pooled effect sizes when the target outcomes were assessed using the same measurements and presented in the same units. Otherwise, standardized mean differences (SMDs) with 95% CIs were applied. The I^2^ statistic was used to report heterogeneity: if P ≤ 0.1 and I^2^ > 50%, heterogeneity was considered significant and analysed using a random-effects (RE) model; if P > 0.1 and I^2^ ≤ 50%, heterogeneity was considered insignificant and analysed using a fixed-effects (FE) model. Sensitivity analysis was conducted to investigate the sources of heterogeneity by excluding studies with high risk of bias and comparing methodological characteristics.

Qualitative data were synthesized using thematic analysis, each code and theme were iteratively developed through a process of both inductive and deductive analysis [34].

## Results

### Study selection

**Fig. 1:**
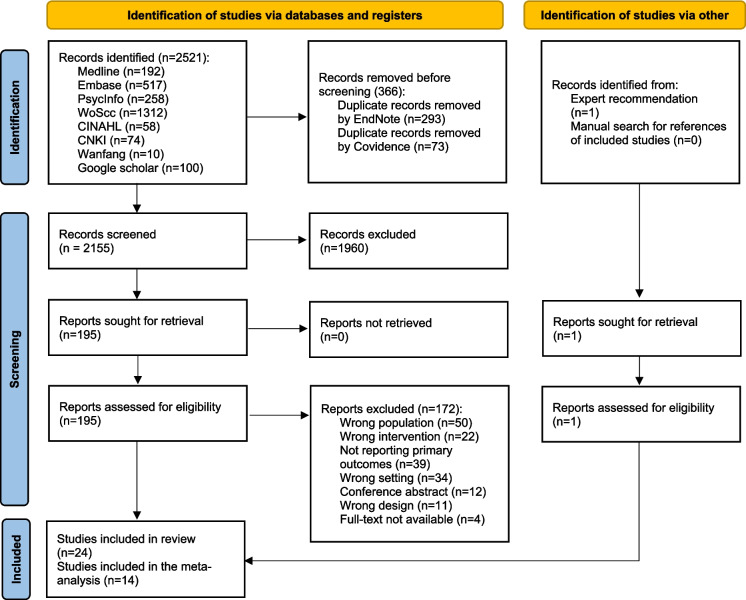
PRISMA-SR flowchart of study selection process

Figure 1 presents the results of the searches, n = 2521. After removing duplicates, 2155 records were included for title and abstract screening, and 195 studies proceeded to full-text review. Additionally, one study was identified by expert recommendation and was included after screening, generating a total of 24 studies: 13 randomised controlled trails (RCTs), three two-arm quasi-experimental studies (QEs), four pre-posttest QEs, two qualitative studies, and two mixed-methods studies. Of the 24 included studies, 22 were published in English and two in Chinese. Only the qualitative components of mixed methods studies were included in this review.

### Characteristics of included studies

The characteristics of the included studies are presented in Table 1.

**Table 1:** Characteristics of the included studies (n = 24)

Study	Design	Setting	Participants	Intervention	Control	Time points	Emerged themes
Alida et al. 2019 Canada	3-arm randomised controlled trial	Research centre in a gym	*Sample size: 41 (IG-1: 12, IG-2: 15, CG: 14)*Age (mean years): IG-1: 68.08, IG-2: 67.20, CG: 67.21*Female: IG-1: 66.67%, IG-2: 73.33%, CG: 85.71%*Health status: inactive	Dance movement training (IG-1)Aerobic exercise training (IG-2)	Routine daily activities	Baseline12-week	–
Amanda et al. 2024 Ireland	Cluster randomised controlled trial	Local community centre and home	*Sample size: 100 (IG: 56; CG: 44)*Age (mean years): IG: 73.73; CG: 74.18*Female %: 87 (87%)*Health status: healthy and active	Music and movement for health (consisted of supervised sessions at a local community centre and a home-based programme combining movement to music and singing)	Usual care	Baseline12-week	–
Dafna et al. 2016 AUS	2-arm randomised controlled trial	Community dance studio	*Sample size: 115 (IG: 60, CG: 55)*Age: IG (48.3% in 60–69, 25% in 70–74, 26.7% in 75 +); CG (60% in 60–69, 20% in 70–74, 20% in 75 +)*Female: IG: 73.3%, CG: 80%*Health status: healthy and active	Social dancing	Home-based self-help walking programme	Baseline3-week8-month follow	–
Ewa et al. 2022 Poland	3-arm randomised controlled trial	Training hall	*Sample size: 37 (IG-1: 13; IG-2: 12; CG: 12)*Age (mean years): 73.3*Female %: total 37*Health status: healthy and active	Folk dance training (IG-1)Balance training (IG-2)	Routine daily activities	Baseline12-week	–
Hee et al. 2019 Korea	2-arm randomised controlled trial	Community centre	*Sample size: 82 (IG: 41, CG: 41)*Age (mean years): IG: 70.5, CG: 71.77*Female %: not stated*Health status: healthy and active	Creative dance	Stretching training	Baseline8-week	–
Josianne et al. 2018 Brazil	3-arm randomised controlled trial	Dance studio and gym	*Sample size: 30 (IG-1: 10, IG-2: 10, CG: 10)*Age (mean years): 65 (IG-1: 66, IG-2: 64, CG: 66)*Female: 100%*Health status: healthy and active	Dancing (IG-1)Walking (IG-2)	Stretching training	Baseline8-week	–
Marcia et al. 2020 Brazil	2-arm randomised controlled trial	Community	*Sample size: 71 (IG: 35, CG: 36)*Age (mean years): IG: 68.6, CG: 70.0*Female %: (IG:97%, CG: 86%)*Health status: healthy and active	Senior dance on risk factors for falls	Single educational class	Baseline12-week	–
Pablo et al. 2023 Chile	2-arm randomised controlled trial	Laboratory	*Sample size: 40 (IG: 21; CG: 19)*Age: 73.26 (IG: 73.91; CG: 72.85)*Female: 100%*Health status: sarcopenia	Group-based dance	Elastic bands training	Baseline12-week	–
Pattanasin et al. 2019 Thailand	2-arm randomised controlled trial	Primary health care units	*Sample size: 78 (IG: 39, CG: 39)*Age (mean years): IG: 66.33, CG: 67.33*Female %: IG: 84.44%, CG: 83.33%*Health status: sedentary	Thai boxing dance	Fall prevention educational booklet	Baseline4-week4-month follow	–
Siddharth et al. 2022 Indian	2-arm randomised controlled trial	Mumbai Thane and Navi Mumbai district	*Sample size: 40 (IG: 20; CG: 20)*Age (mean years): IG: 64.9, CG: 66.4*Female %: 80% (IG: 20, CG: 12)*Health status: healthy and active	Indian folk dance therapy	Conventional therapy exercise	Baseline6-week	–
Wang et al. 2021 China	2-arm randomised controlled trial	Local communities	*Sample size: 44 (IG: 22, CG: 22)*Age: 64.1 (IG: 63.9, CG: 64.6)*Female: 35% (IG: 18%, CG: 17%)*Health status: healthy and active	Modified tap dance programme	Health education lectures	Baseline6-week12-week	–
Zhang et al. 2023 China	2-arm randomised controlled trial	Community in Zhangjiakou	*Sample size: 72 (IG: 36, CG: 36)*Age (mean years): IG: 69.75, CG: 64.94*Female %: 39 (54.2%)*Health status: healthy and active	Square dancing	Routine daily activities	Baseline8-week16-week	–
Defna et al. (2). 2016 AUS	Cluster randomised controlled trial	Retirement villages	*Sample size: 530 (IG: 279, CG: 251)*Age: 39% > 80 years*Female %: 85%*Health status: healthy and active	Social dancing	Routine daily activities	Baseline12-month	–
Anderson et al. 2024 Brazil	2-arm quasi-experimental trial	Londrina, Paraná	*Sample size: 43 (IG: 23, CG: 20)*Age (mean years): IG: 69.6, CG: 67.3*Female %: total 43*Health status: healthy and active	Ballroom dance	Walking training	Baseline12-week	–
Xu 2023 China	2-arm quasi-experimental trial	Community in Lanzhou	*Sample size: 58 (IG: 29, CG: 29)*Age (mean years): IG: 63.06 ± 3.05, CG: 62.23 ± 2.07*Female: 100%*Health status: healthy and active	Guozhuang dance	Health education	Baseline4-week	–
Crystal et al. 2017 US	2-arm quasi-experimental trial	Rural community centre	*Sample size: 23 (IG: 12, CG: 11)*Age: 73.4*Female: 86.96%*Health status: sedentary	Line dancing	Usual care	Baseline8-week	–
Courtney et al. 2022 Canada	Single-arm, pre-/posttest quasi-experimental trial	Young Men’s Christian Association	*Sample size: 107*Age (mean years): 76.1*Female %: 80%*Health status: healthy and active	GERAS dancing	–	Baseline12-week	–
Paolo et al. 2018 Italy	Single-arm, pre-/posttest quasi-experimental trial	Senior social centres	*Sample size: 163*Age (mean years): 70*Female: 123 (75.5%)*Health status: healthy and active	Dance activity	–	Baseline16-week	–
Guo 2014 China	Single-arm pre-/posttest quasi-experimental Trial	Community in Jiaozuo	*Sample size: 120*Age (mean years): 70.5*Female: 61.7%*Health status: low mobility	Fancy dance	–	Baseline12-week	–
Patricia et al. 2023 Canada	Single-arm, pre-/posttest quasi-experimental trial	Young Men’s Christian Association	*Sample size: 23*Age (mean years): 72.5*Female: 75%*Health status: early cognitive or mobility impairments	GERAS dancing	–	Baseline12-week	–
Susan et al. 2017 US	Mixed methods	Adult Wellness Center	Quanti-part:*Sample size: 21 (IG: 10, CG: 11)*Age (mean years): 75.4*Female: 76.2%*Health status: mild cognitive impairmentQuali-part:IG focus group: 7CG focus group: 6	Quanti-part:BAILAMOS dancingQuali-part:2 post-programme focus groups	Quanti-part:Waiting list	–	(1) Enthusiasm for dance(2) Positive aspects of the dance(3) Unfavorable aspects of the dance(4) Physical well-being after dance sessions
Amanda et al. 2021 Ireland	Mixed methods	A venue in community	Quanti-part:*Sample size: 10 (IG: 6, CG: 3)*Age (mean years): 80*Female: 70%*Health status: low mobilityQuali-part:Participants interviews: 5 Stakeholder interviews: 8	Quanti-part:Dance classesQuali-part:Participants interviews and stakeholder interviews	Quanti-part:Singing	–	(1) Pleasure and nostalgia(2) Anticipation and social inclusions(3) Health benefits: physical and emotional well-being
Vanessa et al. 2024 Canada	Qualitative study	Remote (phone or Zoom)	*Sample size: 5*Age (mean years): 76*Female %: 80%	Semi-structured interviews	–	–	(1) Laughter, lightness, and feeling youthful(2) The body comes back to life(3) Confidence can shine(4) Carried away by the music(5) An opportunity to belong(6) Contributing to the neighbourhood spirit
Shawn 2019 US	Qualitative phenomenological study	Private home office	*Sample size: 6*Age (mean years): 79.3*Female %: 100%	6 individual tape-recorded interviews	–	–	(1–1) The positive experience of folk dance(1–2) Negative aspects of folk dance(2–1) Carryover effects(2–2) Social effects(2–3) Emotional effects(2–4) Mental effects—dance and memory(2–5) Physical effects(2–6) Classroom environment factors

#### Participants and setting

A total of 1840 participants were included from 12 countries, with 708 assigned to the dance intervention group and 696 allocated to control groups or other intervention groups, 23 participants were involved in the qualitative interviews. The age of participants ranged from 62 to 80 years, and studies were conducted in Canada (n = 4) [35–38], China (n = 4) [39–42], the United States (n = 3) [43–45], Brazil (n = 3) [46–48], and eight other countries. Fourteen studies reported participants as healthy and active [37, 40–42, 46–55], sedentary in their behaviour (n = 2) [18, 45], having impaired mobility (n = 3) [35, 39, 56], or having a cognitive impairment (n = 2) [36, 43]. Only one study [57] included participants with sarcopenia, and six studies recruited only females. Despite potential mobility limitations or chronic conditions, all participants were able to live independently.

#### Group dance interventions

Nine types of dance interventions were identified: square dance [42], social dance [46, 52, 53], Thai boxing dance [18], tap dance [40], line dance [45], folk dance [41, 54, 55], creative dance [51], GERAS dance [36, 37], and generalized dance movement training without a specific style [35, 39, 47–50, 57]. Most of the dance interventions focused on improving physical function (e.g. balance, postural control, gait, reduction of falls), cognitive function, and psychological status.

The intervention typically included three components: warm-up, core dance training, and relaxation. The core part of the dance varied depending on the intervention goals and dance types, incorporating elements such as rhythmic movements in square dance [42] and Indian folk dance [54], seated and standing dance routines in GERAS dancing [36, 37], coordination training in Guozhuang folk dance [41].

All dance interventions were conducted in group settings, led by professional dance instructors, physical therapists, or trained researchers. Some participants were also required to practice at home to reinforce the intervention effects [37, 50]. The dance sessions were typically conducted in communities or dance studios, each session generally lasting 50–90 min. The overall duration of interventions ranged from 4 to 48 weeks, 50% lasting 12 weeks and 15% lasting 8 weeks. Interventions were tailored in different ways to suit the characteristics of participants, including modifying the music tempo and dance genre [50], adapting the complexity of the dance movements [36, 37, 46, 55], simplifying potentially dangerous movements [40], or tailoring the intervention to participants’ physical condition and adaptability [18, 41, 42, 45, 48].

Diverse materials and resources were employed in the delivery of the interventions, including workshops (e.g., researchers, instructors, public and patient involvement meetings) [50, 52], music and dance movements (e.g., Rock & Roll, Foxtrot, Waltz, Salsa and Rumba) [35, 36, 41, 42, 52, 53], supporting and interactive props (e.g., blankets, colourful scarves, and exercise balls) [35, 39, 51], safety monitoring and recording materials (e.g., instruments and equipment: heart rate monitor-pulsometer and automatic external defibrillator; documents: homework sheets, adverse events calendars, and appointment logbooks) [18, 37, 45, 50, 57], and motivational materials (e.g., small gifts and telephone reminder calls) [18, 42]. The reported range for retention was 66% to 91%, adherence 78% to 89%, attendance 67% to 98.5%, and completion 66.13% to 100%, indicating that most interventions were implemented as planned, with generally good attendance and completion among participants. Details about the dance interventions are presented in Table 2.

**Table 2:** Dance interventions of included studies using the TIDieR checklist (n = 20)

**Study & Intervention**	**Why it was used**	**What materials used**	**What procedure**	**Who provided**	**How it delivered**	**Where it delivered**	**When and how much**	**How well (actual)**		
Dafna et al. (1) 2016 **Social dancing**	To compare (1) changes in age-sensitive cognitive abilities between older adults assigned to ballroom dancing or walking, and (2) changes in exercise capacity and sensorimotor skills between two groups.	*4-h workshop*CDs for music and workbook	*Collection of ballroom dances, Rock and Roll, Foxtrot, Waltz and some Latin*Progressive in terms of motor complexity.*Standardized	Dance teacher	Group	Community dance studio	2 sessions/week32 weeks60 min/session	Retention: 66%Adherence: 78%		
Hee et al. 2019 **Creative dancing**	To investigate the effects of a creative dance program on fitness, functional balance, and mobility in the elderly.To encourage participants create their own movement.	Visual images of spaces	*Warm-up 20 min: engaged in static stretching movements or simple body movement games.*Main activities 45 min: action stage, sequence stage, combination stage*Break 15 min*Cool down 10 min: stretching and relaxation movement	Professional contemporary dance choreographer	Group	Community center for older adults	2 sessions/week8 weeks90 min/session	Average attendance: 98.5%		
Siddharth et al. 2022 **Indian Folk dance**	To explore the effects of Indian folk dance therapy on physical functions and quality of life among elderly.	---	*Warm-up 10 min: free mobility exercises.*Folk dance 45 min: low to moderate level of intensity and consisted of programmed choreography and many more such movements that purely depends on rhythm and beats.*Cool down 5 min: breathing and savasana exercise	---	Group	---	5 sessions/week6 weeks60 min/session	---		
Paolo et al. 2018 **Dancing**	To improve mobility performance and psychosocial function through different dance styles in a group as well as with a partner in a socially engaging environment.	---	*Warm-up 10 min: mobilising exercises*Main phase 40 min: progressive in terms of motor complexity and included different and simple dance movements *Cool-down 10 min: breathing exercises	Graduates and dancing instructors	Pairs or individually	---	2 sessions/week6 weeks60 min/session	100% attended at least 85% of the total dancing sessions.		
Guo 2014 **Fancy dancing**	To observe the effects of dance-based exercise combined with strength training on walking ability.	*5 kg sandbags*Audio-visual productions as training material	*Fancy dance 20 min*Rest 5 min*Weight training 40 min	---	Group	---	afternoon6 sessions/week12 weeks60 min/session	---		
	**Why it was used**	**What materials used**	**What procedure**	**Who provided**	**How it delivered**	**Where it delivered**	**When and how much**	**Tailoring**	**How well (actual)**	
Ewa et al. 2022 **Dance training**	To compare the effectiveness of dance training with balance training on fall risk, physical and cognitive functions.	Heart rate monitor-pulsometer	*Warm-up 10 min*Folk dance training (choreography learning, dance figures, and fitness exercise) 30 min*Static stretching and relaxation exercise.	Qualified dance coach	Group	Training hall	3 sessions/week12 weeks50 min/session	The coach raised the exercise intensity and difficulty every 4 weeks by adding faster music or more complicated dance figures.	Average attendance: 90%	
Marcia et al. 2020 **Senior dancing**	To investigate the effect of Senior Dance on balance, mobility, and cognitive function compared with a control intervention.	None	*Moderate level intensity*Consisted of a range of choreographies, including rhythmic and simple movements with rhythmic folk songs.*Practice at home	Certified instructors who were also physical therapists	Group and individual	——home	*60 min educational class*2 sessions/week12 weeks60 min*Home: 2 days/week, 10-20 min	The intervention not individually tailored to participants.	*Completion: 87%*Average attendance: 67%	
Wang et al. 2021 **Modified tap dance**	To examine the effects of modified tap dance on the improvement of ankle function and postural control in older adults.	---	*Warm-up 10 min*Modified tap dance 45 min*Cool-down 5 min	Senior certified dance instructor	Group	Local communities	3 sessions/week12 weeks60 min/session	Simplified tap dance elements: slower speeds, broken-down movements, substituted dangerous moves	Average attendance: 88.3%	
Zhang et al. 2023 **Square dancing**	To identify whether square dancing can improve participants performance, including physical, psychological, social, and environmental domains.	*Suitable music*Small gifts were rewarded to participants*Face masks were required to were because of OVID-19*Phone call to remind participants	*Warm-up 10 min: simple stretching, joint movement, finger gymnastics*Square dancing 40 min: simple body movements with specific emphasis on rhythm (head rotation, swing upper limb/shoulder movement, chest expansion and lower limb movement, walking forward/backward, turns, and side-to-side movements)*Relaxation 10 min: deep breathing, major muscle groups stretching.	Dance teacher led; trained staff monitored	Group	---	5 sessions/week16 weeks60 min/session	Trained staff monitored older adults' physical condition; stopped dance and provided rest if gait was unstable or tilted.	Completion: 100%	
Anderson et al. 2024 **Ballroom dance**	To improve physical fitness performance	---	*Muscle stretching and body awareness 10 min (light)*Rhythmic and expressive activities 40 min: movements and rhythms proposed in each cycle (moderate)*Relaxing 10 min(light)	Physical education professionals and students	Group	---	afternoon3 sessions/week12 weeks60 min/session	*Participants were allowed to change pace during execution as needed.*All movements were performed in a comfortable range of motion.	*Completion: 76.7%	
Xu 2023 **Guozhuang dance**	To explore the fitness efficacy of Guozhuang dance.	Selected dance tracks and movements	Upper body basic movement; lower body basic dance steps; full-body movement coordination; classic Guozhuang dance routines practice.	---	Group	Dance hall	6 sessions/week4 weeks90 min/session	Based on participants' age, fitness, resilience, and learning background; align Guozhuang dance with exercise load and movement abilities.	---	
Patricia et al. 2023 **GERAS dancing**	To improve gait and reduce fall risk in older adults with early cognitive or mobility impairments.	Standardized curriculum, weekly homework sheets, ProtoKinetics Zeno Walkway for assessments, and music.	*Social movement 10 min*Warm-up 5 min*Structured dance 30 min: combines seated and standing dance routines.*Cool-down 5 min*Review of homework 10min	Certified instructors	Group	YMCA centers	2 sessions/week12 weeks60 min/session	Standardized progressive curriculum, allowing for graded learning and repetition based on participants’ comfort and skill level.	Average attendance: 82.73%	
	**Why it was used**	**What materials used**	**What procedure**	**Who provided**	**How it delivered**	**Where it delivered**	**When and how much**	**How well (planned)**	**How well (actual)**	
Alida et al. 2019 **Dance movement training**	To compare the effects of dance to aerobic exercise	*Themes list*Music*Props (e.g. blanket, colorful scarves, exercise balls).	opening circle, warm up, development, and closure.	Dance therapist or a supervised trainee	Group	Geriatric institution research centre	3 sessions/week12 weeks60 min/session	Attend at least 80% training sessions	Completion rate: 66.13%	
Pablo et al. 2023 **Group-based dance**	To compare the effectiveness of elastic band training regarding group-based dance in older women with sarcopenia.	Automated pressure monitor: participants' vital signs	*Moderate to vigorous, having a heart rate < 120 beats/minute*Warm-up 10 min: low to moderate intensities joint mobility exercises and a low-intensity aerobic programme*Central part 40 min: consisted of dances of moderate to high intensity.*Cool down 10 min: relaxing music and execute dynamic and static flexibility exercises.	---	Group	---	3 sessions/week (Mon, Wed, Fri)12 weeks60 min/session	complete ≥ 85% of all training session	Completion: 86.36%	
Dafna et al. (2) 2016 **Social dancing**	To test social dancing as delivered in the community.	Ballroom- Rock & Roll, Foxtrot, Waltz, Salsa and Rumba. Folkdance- from UK, US, France, Italy, Israel and Greece	Folkdance group were asked to use the Borg Rate of Perceived Exertion in classes 20,40, 60 and 80.	Experiences dance instructors	Face to face	In retirement villages community halls or other room	2 sessions/week12 months60 min/session	Each teacher received a Diary to take attendance and write comments. They were instructed to report the field coordinator on any harm during the trial (such as a fall) and to contact participants who did not showed to class more than 2 weeks to verify continuation.	Mostly delivered as planned; ballroom complicated steps were not taught in slow classes.	
	**Why it was used**	**What materials used**	**What procedure**	**Who provided**	**How it delivered**	**Where it delivered**	**When and how much**	**Tailoring**	**How well (planned)**	**How well (actual)**
Amanda et al. 2024 **Music and movement for health**	To examine the feasibility and acceptability; To present the potential effect and cost effectiveness of the intervention	*Adverse events calendars*Public and patient involvement meeting	*S1: dance sessions (welcome, warm-up, singing, dancing, creative response, cool-down, closing, and a social tea and chat)*S2: home: (combining movement to music and singing), included key movements from the warm-up and cool-down in dance sessions.	dance teacher, music therapist and physiotherapist	Group and home	Local community centre and home	*S1: 90 min/week; 12 weeks*S2: 3 times/week; 12 weeks; 20min each time	Tailored and progressed in line with participants' ability (level of support, tempo, movement sequence complexity) and preferences (music and dance genre).	*Minimum retention rate: 80%*Minimum average attendance: S1: 65%; S2: three times per week.	*Retention: 91%*Average attendance: 71%*80 %participants attending at least 65 % of the sessions.*Adherence: 89 %
Josianne et al. 2018 **Dancing**	To compare the effects of dancing with walking on cardiovascular risk and functionality of older women	---	*Warm-up: week1-3: learning posture, technique and coordination; week4-6 and week 7-8: Performing exercises*Across-the-floor: week1-3: learning isolated steps; week4-6 and week7-8: performing sets already learned*Choreography: week1-3: learning steps put together; week4-6: adding on new steps; week7-8: completing the full chorography*Show and cool-down: week1-3: performing the choreography; week4-6 and week7-8: repeating the choreography.	Specialized instructors	Group	Dance studio	3 sessions/week8 weeks60 min/session	The warm-up ~55% VO2peak (95–115 bpm), the across- the-floor ~62% VO2peak (115–125 bpm), choreography ~63% VO2peak (128 bpm), and the show ~69% VO2peak (128 bpm).	Being mandatory to complete 24 dancing sessions.Anticipated an attrition rate: 20%.	Compliance: over 95%.
Courtney et al. 2022 **GERAS dancing**	To examine the effect of GERAS dance on physical function and the level of frailty and cognitive impairment.	*Homework sheets for non-class days*Standardized training session for the dance instructors*Monthly implementation meetings to share resources and strategies.	*Introduction/socialization 10 min*Warm-up 5 min *Structured dance curriculum 30 min*Cooldown 5 min*Review of weekly homework 10 min	Dance instructor	Group and homework	Sites of YMCA	2 sessions/week12 weeks60 min/session	Dances are taught in both the seated and standing positions and gradually increase the time spent standing.	Homework sheets for non-class days, target: 10 min daily	*Over 90% attended at least half of the classes*Average attendance: 75%*Fidelity: 100%
	**Why it was used**	**What materials used**	**What procedure**	**Who provided**	**How it delivered**	**Where it delivered**	**When and how much**	**Tailoring**	**Modification**	**How well (actual)**
Pattanasin et al. 2019 **Thai Boxing dance**	To evaluate the effects of Thai Boxing dance on static and dynamic balance performance, and functional fitness.	*Appointment logbook*Telephone remind call	*Warm-up 10 min: light load at a comfortable speed, upper and lower limb stretching.*Thai boxing dance 30min: specific patterns, slow pace at weeks 1-2, faster pace at weeks 3-4. Each pattern performed 10 times.*Cool down 10 min: walking and muscle stretching similar to the warm-up period.	Trained instructor with four assistants	Group	Primary health care units	3 sessions/week4 weeks50 min/session	Participant could rest at any time during the dance period if they had any uncomfortable symptoms or felt tired.	Modified from Kantamara (2010) and Tantiwiboonchai et al. (2017).	Completion: 100%
Crystal et al. 2017 **Line dancing**	To assess the effects of line dancing on balance, muscle strength, lower extremity function, endurance, gait speed, and perceived mobility limitations.	*Safety monitoring: Borg Rating of Perceived Exertion Scale*Comfortable shoes*Automatic external defibrillator (AED)*Emergency contact information.*Opportunity for questions.	*Warm-up 10 min: seated stretching*Dancing 40 min: learning new dances, practice time, a break and reviewing dances learned in previous classes. 8 specific choreographed dance routines*Cool-down 10 min	Dance instructor	Group	Community center	2 sessions/week8 weeks60 min/session	Participants rated exertion from 6 to 20 and were told to rest if feeling dizzy, weak, short of breath, or had chest pain.	Modifications were recorded each session. The instructor and PI observed participants and offered simpler movements if needed.	*Completion: 80%

Not including Tailoring, Modification and How well planned. Because the studies in this table do not provide the relevant information

### Outcomes and measurements

#### Primary outcomes

##### Muscle mass

Only one RCT [35] assessed the effect of dance intervention on muscle mass, so meta-analysis was not possible. As reported in the original study, participants in the dance group showed a significant increase in appendicular lean body mass after training (F (1,38) = 4.70, P = 0.04), but no significant group difference or interaction effect was observed. The medium effect size (η^2^p = 0.11), which represents the proportion of variance explained by the intervention, further supports the potential of training to improve muscle mass.

##### Muscle strength

Muscle strength was assessed in seven RCTs [18, 35, 42, 48, 51, 53, 57] and three QEs [41, 45, 46]. The included studies used different measurement tools and assessed muscle strength in various body regions. Specifically, four studies [35, 42, 46, 57] measured upper limb strength using handgrip strength tests, and two studies [41, 51] assessed both upper and lower limb strength using the 30 s chair arm curl test and the 30 s chair stand test, respectively. Additionally, assessments of hip, knee, and ankle strength were conducted in four studies [18, 45, 48, 53] using isokinetic dynamometers, push–pull dynamometers, and manual hand-held dynamometers.

Due to differences in measurement tools and data types, only four RCTs [18, 42, 53, 57] were included in the meta-analysis. Subgroup analyses were conducted by categorizing muscle strength into grip strength and knee extensor strength (Fig. 1 in Supplementary Materials B). High heterogeneity was observed in the pooled results (I^2^ = 72%), suggesting variability in study designs and measurements. For grip strength, the combined effect size was −0.08 (95% CI: −0.54, 0.39, *P* = 0.75), indicating no significant difference and low heterogeneity (I^2^ = 32%). For knee extensor strength, the combined effect size was 0.29 (95% CI: −0.44, 1.02, *P* = 0.43), also showing no significant improvement but with high heterogeneity (I^2^ = 89%). When comparing intervention effects, dance showed potential benefits compared to education (SMD = 0.70, 95% CI: 0.24, 1.16), but no significant effect was found compared to usual care (SMD = −0.05, 95% CI: −0.22, 0.12). Overall, the meta-analysis indicated that the dance intervention did not significantly improve muscle strength (SMD = 0.11, 95% CI: −0.27, 0.49, *P* = 0.56).

##### Physical performance–5 times Sit-To-Stand test (5STS)

**Fig. 2:**
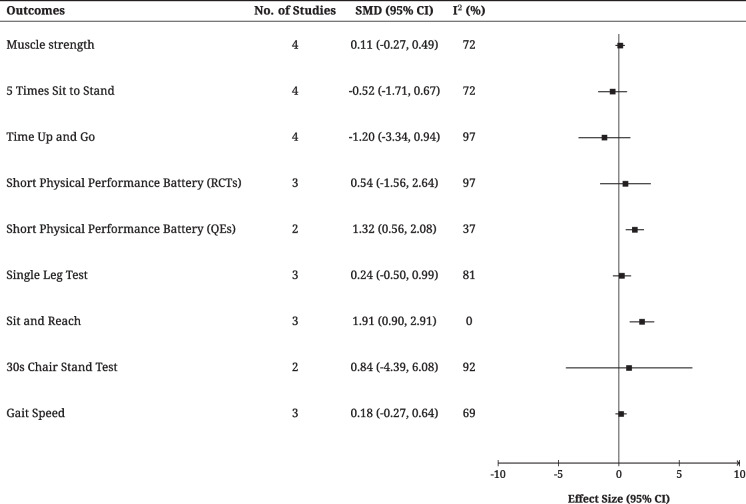
Meta-analysis summary of the included primary outcomes (muscle strength and physical performance). Note: Detailed forest plots for each outcome are provided in the Supplementary Materials

Eight studies used the 5STS to evaluate physical performance [58], including six RCTs [40, 42, 47, 48, 52, 53], one two-arm QE [46], and one pre-post test QE [37]. Both QEs demonstrated that participants required less time to complete the test after the dance intervention, with the improvement in physical performance being statistically significant (*p* < 0.01). Four RCTs [40, 47, 48, 53] were included in the meta-analysis, showing a favorable trend for dance but without statistical significance and with high heterogeneity (72%). In subgroup analyses, dance showed certain advantages compared to other forms of exercise and education, although these differences were not statistically significant. Among the included studies, three studies [40, 47, 48] favoured dance, but only one [53] achieved statistical significance. After 12 months of dance intervention, no improvement was observed in 5STS performance compared to usual care (Fig. 2).

##### Dynamic balance–Time Up and Go test (TUG)

Eight studies assessed physical performance using the TUG test [59], comprising seven RCTs [18, 35, 48, 50, 51, 55, 57] and one pre-post test QE [49]. Among them, five studies reported that dance training significantly reduced the test completion time, indicating improved physical performance [18, 48, 49, 51, 55]. In terms of meta-analysis, four RCTs [50, 51, 55, 57] were included. The combined results showed high heterogeneity (97%), indicating some variability among studies. Despite this, a significant overall effect of dance intervention was observed (MD = −1.20, 95% CI: −1.34, −0.94). In the subgroup analysis, dance showed a significant improvement in dynamic balance compared to usual care. However, when compared to other exercise interventions, the difference was not statistically significant (Fig. 2).

##### Short Physical Performance Battery (SPPB)

Six studies assessing SPPB [60] were included in the analysis: three RCTs [42, 50, 53], two two-arm QEs [45, 46], and one pre-post test QE [37]. To minimize heterogeneity caused by differences in study design, separate meta-analyses were conducted based on study type (Fig. 2). In the meta-analysis of the three RCTs [42, 50, 53], all control groups received usual care. However, high variability (I^2^ = 97%) was observed across studies, potentially due to differences in sample size, intervention protocols, and participant characteristics. Only one study [42] demonstrated positive effects of the dance intervention, while the overall effect was not significant (MD = 0.54, 95% CI: −1.56, 2.64). In the meta-analysis of the two two-arm QEs [45, 46], a significant improvement (MD = 1.32, 95% CI: 0.56, 2.08) and low heterogeneity (I^2^ = 37%) was observed, suggesting a beneficial effect of dance interventions and relatively consistent results. However, the study by Crystal et al. [45] reported a larger effect size (MD = 2.42) with a wide 95% CI (−0.84, 3.24), reflecting some uncertainty due to small sample size and different controls.

##### Static balance–Single Leg Test (SLT)

Four studies [47, 48, 50, 54] utilized SLT to measure static balance [61], with two [47, 54] reporting significant positive effects of dance on physical performance (*P* < 0.05). Because of the lack of detailed information on standing conditions (e.g., left vs. right leg or eyes open vs. closed) in some studies, SMD was used to standardize the data. The variation in control groups, including walking training [48], education, and usual care, potentially contributed to the high heterogeneity (I^2^ = 81%). In terms of effect size direction, the study by Josianne et al. [48] showed weaker performance in the dance group compared to the walking group, although the results did not reach statistical significance. While dance interventions demonstrated a positive trend in some studies, particularly when compared to education [47], the overall meta-analysis results for the SLT were not statistically significant (SMD = 0.24, 95% CI: −0.50, 0.99) (Fig. 2).

##### Flexibility–Sit and Reach Test (SRT)

Three RCTs [18, 48, 51] assessed body flexibility using SRT [62]. The meta-analysis of these studies showed that all effect sizes favoured the dance intervention (I^2^ = 0), the pooled mean difference was 1.91 (95% CI: 0.29, 2.91, *P* = 0.02) (Fig. 2). Subgroup analysis was conducted due to the variation in the control groups. Two studies [48, 51] compared dance to stretching training (I^2^ = 0). However, the results were not significant, suggesting that dance intervention may not be significantly superior to stretching (95% CI: −0.67, 5.44). In contrast, one study [18] compared dance intervention to education, showing a significant advantage for dance intervention (MD = 1.85, 95% CI: 0.77, 2.91, *P* = 0.007). Overall, despite differences in control conditions, the meta-analysis supports the positive effect of dance intervention on older adults’ flexibility measured by SRT.

#####  30 s Chair Stand Test(30CST)

Three studies included the 30CST [63] (two RCTs [50, 51] and one two-arm QE [41]). The QE study [41] showed that the Guozhuang dance intervention led to better performance compared to the education group. The meta-analysis of the two RCTs [50, 51] showed clearly opposing effect sizes and considerable heterogeneity (I^2^ = 92%) (Fig. 2). Compared to the stretching group, the study by Hee et al. [51] showed a significant effect of dance intervention (MD = 3.53, 95% CI: 1.73, 5.49). In contrast, Amanda et al. [50] found no significant result, and the dance group showed poorer performance than the usual care group (MD = 1.31, 95% CI: −1.03, 3.91). The overall pooled results indicated no statistically significant difference between the dance and control groups (MD = 0.84, 95% CI: −4.39, 6.08, *P* = 0.67).

##### Gait speed

Gait speed was reported in 14 studies (nine RCTs [35, 42, 47, 51–55, 57], two two-arm QEs [45, 46], and three pre-post test QEs [36, 37, 39]), with 71.4% showing positive changes when compared to controls or pre-intervention status [35–37, 39, 42, 45, 47, 51, 54, 55]. The final pooled results indicated that dance interventions had no effect in improving gait speed (MD = 0.18, 95% CI: −0.27, 0.64, *P* = 0.04, I^2^ = 69). Similarly, there was no significant improvement in subgroup analysis when compared to the usual care group (MD = −0.04, 95% CI: −0.21, 0.13, *P* = 0.93, I^2^ = 0) (Fig. 2).

#### Secondary outcomes

##### Psychological outcomes–cognition

**Fig. 3:**
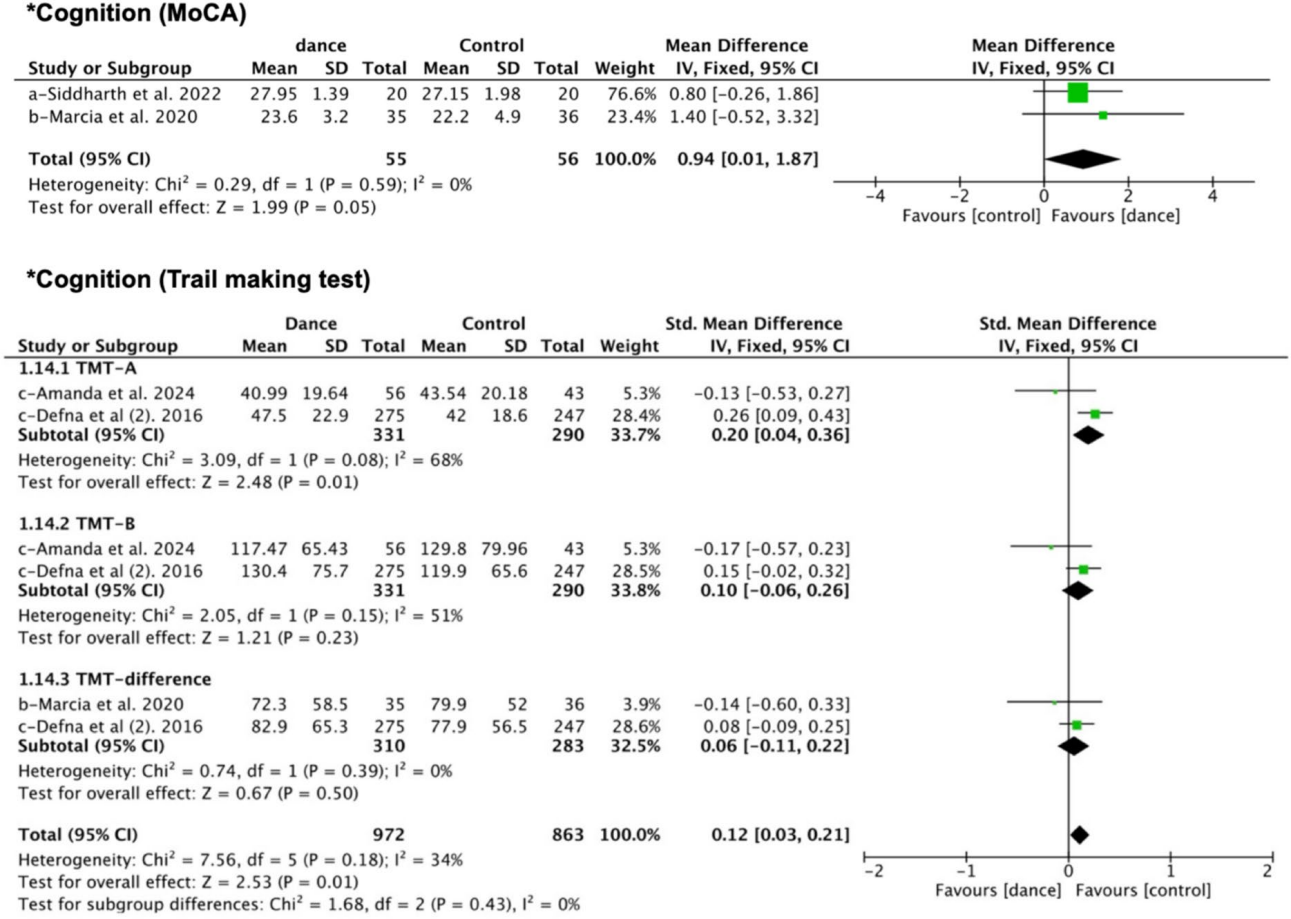
Forest plot of cognition (*statistically significant; a, exercise control; b, education control; c, usual care [routine daily activities])

Two RCTs [47, 54] reported effects using the Montreal Cognitive Assessment test (MoCA) [64], and the data were suitable for meta-analysis. The pooled analysis suggested that dance intervention had a statistically significant effect on improving cognitive function (MD = 0.94, 95% CI: 0.01, 1.87, *P* = 0.05, I^2^ = 0). Three RCTs [47, 50, 53] used Trail Making Test (TMT) to evaluate cognition. The subgroup analysis for TMT-A (SMD = 0.20, 95% CI: 0.04, 0.36, I^2^ = 68) slightly favored the dance group, with a statistically significant difference. The effect sizes for TMT-B and the TMT B-A difference showed slight positive trends but were not statistically significant (Fig. 3). Overall, the combined results indicated a small but statistically significant positive effect (SMD = 0.12, 95% CI: 0.03, 0.21, I^2^ = 34), suggesting that dance intervention may contribute to improving cognitive functions, including processing speed, task-switching, and executive function.

##### Social outcomes

Six of the included studies evaluated social outcomes using various measurements, including the Lubben Social Network Scale (LSNS) [35, 49, 52], the Berkman-Syme Social Network Index (SNI) [50], the UCLA Loneliness Scale [50], the Frailty Scale (FI-35) [42], and the Self-Rated Health Measurement Scale (SRHMS) [41]. The results showed positive trends in social contact and social networks in some studies [42, 49]. We did not perform a meta-analysis due to the heterogeneity of measurements and indicators. Detailed effect directions are presented in Table 3.

**Table 3:** Outcomes of included quantitative studies (n = 20)

Study	Sarcopenia (MM, MS, PP)	Body composition (excluding MM)	QoL	Social	Mental	Economic	Adverse outcomes
Alida et al. 2019	MM: Appendicular lean body mass ⊜MS: HGS ⊜PP: TUG ⊜, 10-m WT ⊜	BMI ⊜	SF-12 physical ⊜; SF-12 mental ⊜	Social network: LSNS ⊜	*Cognition: MoCA ⊜*Depression: GDS ⊜*Anxiety: state trait anxiety inventory, STAI ⊕ *Mental health: mental health continuum short form ⊜	–	–
Amanda et al. 2024	PP:*TUG ⊜*Lower extremity strength and endurance: 30CST ⊜*Static postural control: SLT ⊜*Lower limb function: SPPB ⊜	–	EQ-5D-5L ⊜; IGECAP-O ⊜;	*Social isolation: Berkman-Syme social network index, SNI ⊜*Loneliness: UCLA loneliness scale ⊜	*Cognition: TMT ⊜*Mood: GDS-5 ⊜	*Quality Adjusted Life Years, QALY*Incremental Cost-Effectiveness Ratio, ICER	3 pains
Dafna et al. (1). 2016	PP: 6 min WT ⊜, 5STS ⊜, 6-m WT ⊜	–	–	Social network: LSNS ⊜	Cognition: TMT ⊜	–	–
Ewa et al. 2022	PP: TUG ⊕, 6 min WT ⊕	–	–	–	Cognition: Determination test, DT ⊕	–	–
Hee et al. 2019	MS:*Lower body: 30CST ⊕ *Upper body: 30 s arm curl test ⊕ PP:*Lower body flexibility: SRT ⊜*Upper body flexibility: back scratch test ⊜*Balance: BBS ⊕ *Mobility: TUG ⊕; dynamic gait index ⊕; 10-m WT ⊕	–	–	–	–	–	–
Josianne et al. 2018	MS: Isometric and Isokinetic Knee Extension ⊜PP:*Static balance: SLT ⊕ *Dynamic balance: TUG ⊕ *Flexibility: SRT ⊜*Ability to seat and stand: 5STS ⊜	Body weight ⊜; waist and hip circumferences ⊜; visceral adipose tissue ⊜; quadriceps thickness ⊜	–	–	–	–	Light muscle soreness
Marcia et al. 2020	PP:*Balance: SLT eyes closed ⊕ *Mobility: standing balance ⊕, 5STS ⊕, 4-m WT ⊕	–	–	–	Cognition: TMT ⊜; MoCA ⊜	–	Fall
Pablo et al. 2023	MS: HGS ⊕, leg strength (ten-point RPE) ⊕ PP: TUG ⊕, 4-m WT ⊕	Fat mass ⊜; Fat-free mass ⊕	–	–	–	–	None
Pattanasin et al. 2019	MS: Lower limb: hip flexors ⊕, hip extensors ⊕, knee flexors ⊕, knee extensors ⊕, ankle dorsiflexors ⊕, ankle plantar flexors ⊕ PP:*Dynamic balance: TUG ⊕ *Static balance: Romberg test ⊕ *Back and leg flexibility: SRT ⊕ *Agility: 8-foot up and go test ⊕	–	–	–	–	–	None
Siddharth et al. 2022	PP:*Balance: Fullerton advanced balance ⊕; SLT eyes closed ⊕ *6-min WT ⊕	–	SF-36 physical ⊕; SF-36 mental ⊕	–	Cognition: MoCA ⊕	–	–
Wang et al. 2021	PP:*5STS ⊕ *Range of motion: left ⊜ and right ankle dorsiflexion ⊜, left ⊕ and right ankle plantar flexion ⊕ *Postural control: 3 standing tests ⊜	–	–	–	–	–	None
Zhang et al. 2023	MS: HS ⊕ PP: SPPB ⊕, 10 s standing balance ⊜, 5STS ⊕, 4-m WT ⊕	–	–	Social contact: FI-35 ⊕	*Depression: GDS ⊕ *Emotion: FI-35 ⊕ *Cognition: FI-35 ⊕	–	None
Dafna et al. (2). 2016	MS: leg strength (knee extension) ⊜PP:*SPPB ⊜, 5STS ⊜, gait speed ⊜	–	SF-12 physical ⊜; SF-12 mental ⊜	–	Cognition: TMT ⊜	–	–
Anderson et al. 2024	MS: HGS ⊜PP:*Lower limb: SPPB ⊜, 10 s standing balance, 4-m WT ⊜, 5STS ⊕ *Agility and dynamic balance: AGI test ⊜*6 min WT ⊜	–	–	–	–	–	–
Xu 2023	MS:*Upper body: number of dumbbell lifts ⊕ *Lower body: 30CST ⊕ PP:*Mobility: 2.4-m rise and walk test ⊜*Flexibility: Chair Bend Test ⊜, Back Hands Test ⊜	–	–	*Social Adaptation: SRHMS ⊜*Social Resources: SRHMS ⊜*Social Role: SRHMS ⊜	Positive happiness ⊕ Mental worries ⊕ Fatigue ⊕ Positive emotions ⊜Psychological symptoms and negative emotions ⊕ Cognitive functioning ⊜General health ⊜	–	–
Crystal et al. 2017	MS: Knee extensors strength ⊕ Knee flexors strength ⊜PP:*Balance: BBS ⊕ *SPPB ⊕ *Endurance: 400 m walk ⊕ *Perceived mobility limitations ⊜	–	–	–	–	–	–
Courtney et al. 2022	PP:*SPPB ⊕, 10 s standing balance ⊜, 4-m WT ⊕, 5STS ⊕	–	–	–	–	–	None
Paolo et al. 2018	PP:*TUG ⊕ *Rapidly changing direction: four square step (FFS) ⊕	–	SF-12 physical ⊕; SF-12 mental ⊕	Social network: LSNS ⊕	–	–	–
Guo 2014	PP:*Stride width ⊕ *Balance beam walking ability ⊕ *500-m walking time ⊕	–	–	–	–	–	–
Patricia et al. 2023	PP:*Gait: gait speed ⊕, stride length ⊕, stride width ⊜	–	–	–	–	–	–

*MM*, muscle mass; *MS*, muscle strength; *PP*, physical performance; *QoL*, quality of life; *HGS*, handgrip strength; *TUG*, time up and go test; *30CST*, 30 s sit to stand test; *5STS*, 5 times sit to stand test; *WT*, walking test; *SRT*, sit and reach test; *SPPB*, Short Physical Performance Battery; *SLT*, single leg test; *SSB*, standing static balance; *BBS*, Berg balance scale; *SMM*, skeletal muscle mass; *FFM*, free fat mass; *FM*, body fat mass; *SF-12*, 12-Item Short Form Health Survey; *SF-36*, 36-Item Short Form Health Survey; *EQ-5D-5L*, EuroQol 5-Dimension 5-Level; *IGECAP-O*, Instrument for Geriatric Comprehensive Assessment and Plan for Older Adults; *LSNS*, Lubben Social Network Scale; *TMT*, trail making test; *MMSE*, Mini-Mental State Examination; *SRHMS*, Self-Rated Health Measurement Scale; *FI-35*, Frailty Index-35; *GDS*, geriatric depression scale; *MoCA*, Montreal Cognitive Assessment ⊕, positive change; ⊖, negative change; ⊜, no change

##### Body composition

Three studies [35, 48, 57] assessed body composition (excluding muscle mass), using indicators such as BMI, body weight, waist and hip circumference, visceral adipose tissue, quadriceps thickness, fat mass, and fat-free mass (Table 3). Only fat-free mass showed a positive improvement trend [57]. Meta-analysis was not performed.

##### Quality of life

Five studies (four RCTs [35, 50, 53, 54] and one pre-post test QE [49]) evaluated quality of life (QoL) using different tools (e.g., SF-12, SF-36). The results using the SF-36 indicated significant improvements in both physical and mental health dimensions [54]. However, the findings using the SF-12 were inconsistent in three studies [35, 49, 53]. Overall, a potential positive trend in improving quality of life was observed. Table 3 presents the specific effect directions.

##### Adverse events

Two kinds of intervention-related adverse events were identified: pain [50] and light muscle soreness [48]. Three cases of pain occurred during home-based dance training, caused by previous injuries, incorrect or excessive training [50]. Most studies did not mention information regarding adverse events, while five studies clearly stated that no intervention-related adverse events were reported [18, 37, 40, 42, 57].

##### Economic outcomes

One study [50] conducted an economic analysis using Quality-Adjusted Life Years (QALY) and Incremental Cost-Effectiveness Ratio (ICER). The results suggested that the intervention demonstrated potential cost-effectiveness [50]. However, medication costs were not included in the analysis, and the lack of national unit cost data and missing data may have influenced the results.

#### Qualitative findings

Four studies explored older adults’ views and experiences of dance interventions using interviews and focus groups. The four studies [38, 43, 44, 56] identified three themes: psychosocial wellbeing, physical wellbeing, and cognitive wellbeing. Each theme consisted of several sub-themes, reflecting the multidimensional impacts of dance interventions (Table 4).

**Table 4:** Thematic analysis of participants’ views and experiences of group dance interventions (N = 4)

Themes	Subthemes	Inductive coding	Example quotation
Psychosocial wellbeing	Enjoyment	Positive emotions	• Exercise is just exercise, but you like the little bit of fun and have a laugh
		Positive emotions	• And wherever there is dance there is happiness
		Positive emotions	• It’s camaraderie, with the other folks that were there. It’s doing the exercises [that] is a lot of fun. I enjoy myself
		Positive emotions	• The music is just so beautiful. So, it’s not work, it’s not exercise for me. It is a fun thing
		Relaxed	• …refreshed and relaxed, encourages us toward lightness
		Motivation	• If you sit at home all day, you’re going to fall away
		Motivation	• Dance has restored a bit of hope that, physically you’ve still got some ability
		Playful	• There is a playful aspect which has not really been a part of my life since a very young age so, that’s kind of fun to cultivate, to see coming out of other people. We’re not [said mockingly] ‘second childhood’ but [laughs] we can play
		Nostalgia	• It takes me back, bringing back memories, and I think it’s lovely
		Enjoy	• I like Greek dancing…I like Israeli dancing…I like the energy level and the dance steps
		Enjoy	• I was going to [do the recorded videos], but never did get around to it... I should be doing it
		Confidence	•.. pleased to be moving again... and thankful that to my body that it will still move
		Confidence	• Oh my gosh Anna, I’m surprised. You did it! I feel good. I feel good…yeah!
		Self-expression	• movements can be fluid, and there’s more space I suppose for some self-expression
		Energetic	• I feel like I have more energy each day…dancing makes you feel more energy, more energized
		Energetic	• I feel better after I finish a class, it gives me energy for the rest of the day
		Sense of accomplishment	• It is quite an accomplishment. It is like doing a crossword puzzle that you completed. Dancing creates a feeling of well-being and happiness
	Social connections	Peer support	• Oh, we encourage each other to go [to the dance class]
		Peer support	• We have become very close emotionally like family…when I was recently ill; I could not believe how much support I got from all my dance mates. They called, visited me, brought me flowers, and it was amazing. I was really quite surprised because I do not see myself as a very likable person…so this was the first time I realized, ‘Oh my god, people really care about people
		Group connection	• It’s nice to have a group to come back to, and it’s nice to have a group that you can move with instead of explaining yourself
		Group connection	• We can share things, we can ask for help, we can, you know it’s nice just to chit-chat
		Group connection	• And I thought that it would be wonderful to be able to see some of the other people in the community that were in my age group... I just thought it was an important community activity that I needed to be involved in
		Group connection	• You feel much more part, and probably more confident if you’re in a group than if you do it by yourself
		Group connection	• Another participant newer to the group described it as “really warm and welcoming”
		Social interaction	• It’s good to make other acquaintance because when you’re in your 80 s many of your friends die, and this is, a new opportunity to be a part of a community
		Social interaction	• I’ve met new friends in folk dance, and I think the social part of it has been good. We enjoy the music together
		Social interaction	• This [class] is not just an exercise class; it also has the social aspect along with it
		Carryover effect	• It’s a friendly group…and you do other things with them outside of class…so it carries over beyond the class I think
	Facilitators to psychosocial/cognitive wellbeing	Rhythm of music	• I think music and dance I find a link within my body or my mind... So I think there must be some connection there that, the music improves my movement
		Enjoy music	• for me, you know, listening to music and you float with your music and I’m on cloud when I dance
		Enjoy music	• When I’m dancing, I’m feeling better, and I hear the music…its positive. I think it does make me feel better physically and emotionally
		Role of music	• I find that movement to music is much more [conducive] than making a space in my condo and just doing some exercises
		Music resonance	• I enjoy moving to music, but I really enjoy moving to music that I can identify with... we are all about the same age group so we all can identify with things like the Beatles, and whatever else was in vogue in the 60 s and 70s
		Good instructor	• We have really good instructors…they explain everything really well…and they make it fun!
		Patient instructor	• …very patient and she loves to teach…she does not mind going over steps until you get it. She is really calm in the way she interacts with everyone in class
		Patient instructor	• She is very patient. When I started folk dance I was very new…I never danced before…she has a wonderful smile
	Barriers to psychosocial/cognitive wellbeing	Frustrating	• It’s a little frustrating when I can’t pick up the new dance routine. I mean, it’s frustrating because I can’t pick up the routine as quickly as I remember picking them up when I was younger
		Self-doubt	• Oh Anna, you are dumb. Why don’t you do it? Why can’t you learn it? Why can’t you remember?
		Challenging	• …when it gets to complicated, to many steps, I will take a break to remind me of my limits
Physical wellbeing	Mobility & balance	Mobility	• You’re moving a bit better; you’re walking a bit better because of the class
		Balance	• I think dance has strengthened my balance and posture through the movement, the asymmetric moving, and through the rhythm
		Balance	• …during the dance routine you sway back and forth, you change the center of gravity…It does help with balance
	Body control	Body control	• I’ve done nothing for a couple of years, and so when she starts, I guess the body comes back to life or something
	Health improvements	Medication reduction	• I used to take medication for my diabetes but now the doctor stopped my medication. I attribute this to my dancing and walking…It’s because I exercise…My doctored joked with me and asked ‘can you dance more?’
		Cardiovascular health	• I went to the cardiologist last week, he found I lost six pounds in the last year…I also attribute this to dance and exercise
Cognitive wellbeing	Memorization	Memorization	• I like folk dance so much because I realize how it works my brain by memorizing steps. I like to learn in new ways by remember things…It gives me good self-confidence
		Memorization	• I think it helps my brain. It’s similar to knitting…knitting and dancing are really similar…because in folk dance there are steps that are repeated…knitting is the same way, if you want to put some pattern in it, you have to memorize it

Psychosocial wellbeing included four sub-themes such as enjoyment, social connections, and both facilitators and barriers to well-being. Participants reported feeling happier, more energized, and more connected to others through dance. Appropriate music and patient instructors made the dance experience more enjoyable, but complex dance routines were reported as challenging, contributing to self-doubt and frustration. Physical wellbeing included three sub-themes, including improvements in mobility, balance, and body control. In terms of overall health, participants reported reduced reliance on medication and a lower cardiovascular risk. Cognitive wellbeing centred on one sub-theme, which focused on memorization, as participants emphasized the challenges of learning and remembering dance routines.

### Risk of bias assessment

**Fig. 4:**
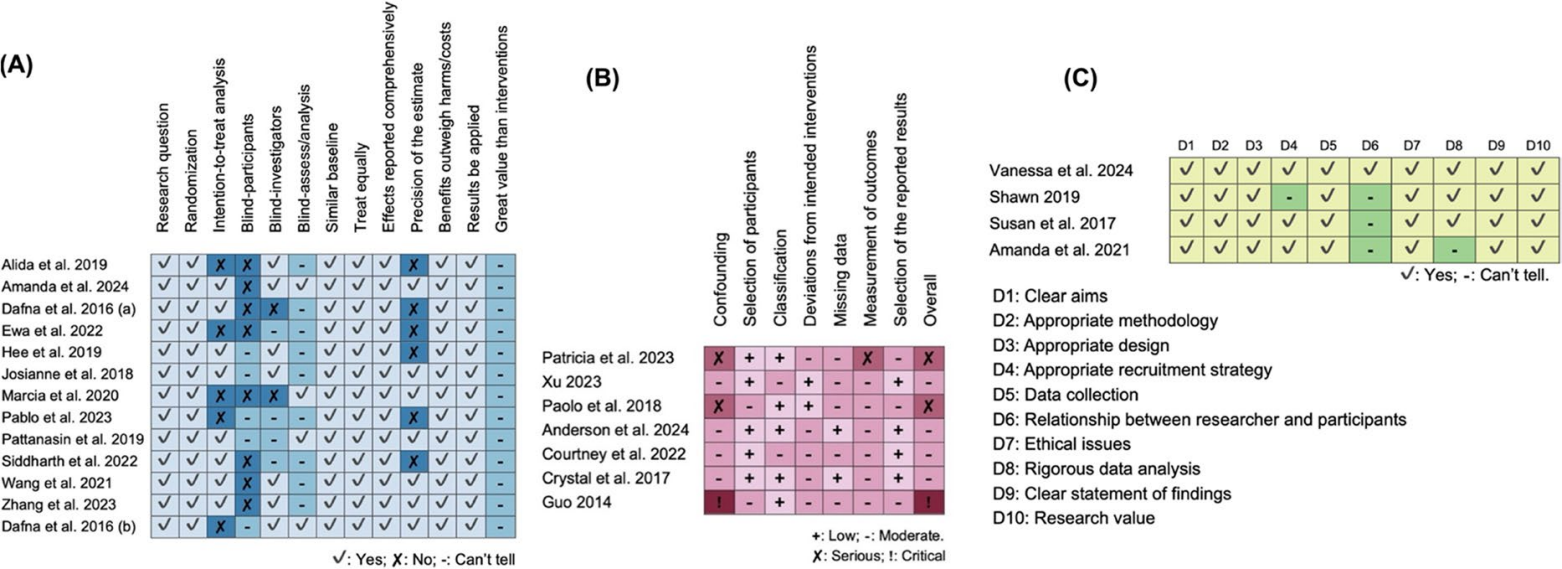
Results of the risk of bias assessment: (A) CASP Checklist for Randomised Controlled Trials (N = 13); (B) Risk Of Bias In Non-randomized Studies – of Interventions (ROBINS-I) tool (N = 7); (C) CASP Checklist for Qualitative Research (N = 2) or Qualitative Component (N = 2)

Most RCTs showed a high risk of bias in one or two domains, mainly related to blinding (including participants, investigators, and analysis) and the precision of the estimates. For blinding, eight studies [35, 40, 42, 47, 50, 52, 54, 55] did not blind participants and nine studies [35, 40, 42, 48, 51, 52, 54, 55, 57] did not provide information on whether the analyses were blinded or not. However, all studies were low risk for randomization, similarity of baseline characteristics, comprehensiveness of outcome reporting, benefits and the applicability of results, which reduces the potential influence of confounding factors (Fig. 4A). A domain-level summary of the CASP assessments for RCTs is provided in supplementary materials C (eTable 1).

All included QE studies showed a moderate to critical risk of bias in confounding and outcome measurements, with some studies [36, 49] showing serious risks and one study [39] reaching a critical risk level. Five studies were at low risk of bias regarding participant selection [36, 37, 41, 45, 46] and classification of interventions [36, 39, 45, 46, 49]. Five studies showed a moderate risk of bias in intended intervention [36, 37, 39, 45, 46] and missing data [36, 37, 39, 41, 49]. Outcome measurement and selection also presented a moderate risk of bias. Overall, the risk of bias in most QEs ranged from moderate to critical levels (Fig. 4B). The ROBINS-I summary is presented in supplementary materials C (eTable 2).

The qualitative studies (n = 2) or studies with a qualitative component (n = 2) indicated low risk across seven domains (aims, methodology, study design, data collection, ethics, statement of findings, and research value). Three studies did not report the relationship between researchers and participants [43, 44, 56]. Recruitment strategies were not clearly reported in one study [44], and data analysis procedures were insufficiently described in one study [56] (Fig. 4C). A summary of the CASP qualitative assessments is included in supplementary materials C (eTable 3).

### Publication bias

In view of the small number of included studies contributing to each meta-analysis (2–4 studies per outcome), it was not meaningful to examine publication bias statistically using measures such as the Egger test and funnel plot analysis. Nevertheless, the potential influence of publication bias cannot be excluded.

## Discussion

This review has shown that dance interventions including square dance, folk dance, and social dance are used increasingly to improve the physical, psychosocial, and cognitive health of older adults. These dance interventions often incorporate their respective cultural contexts [18, 42] and were offered by professionals or trained researchers [37, 46, 50].

Regarding the effectiveness of group dance in preventing and treating sarcopenia, empirical evidence is limited. To rigorously address this evidence gap, we examined studies that reported sarcopenia core outcomes, including muscle mass, muscle strength, and physical performance, rather than limiting our inclusion to studies specifically targeting sarcopenia. This was because sarcopenia-specific studies are currently limited in the literature, which is an important finding. Only one study explicitly recruited participants with a sarcopenia diagnosis, and none designed the dance intervention specifically for this condition. As a result, most available evidence relates to physical performance, with far fewer assessments of muscle mass or strength. Additionally, variation in outcome measures and assessment methods also created challenges in synthesising findings.

Despite these limitations in the available evidence, we conducted a meta-analysis to evaluate the effects of group dance on core sarcopenia-related outcomes and provided a descriptive interpretation of the findings.

The meta-analysis showed significant improvements in Short Physical Performance Battery (SPPB), Sit and Reach Test (SRT), Montreal Cognitive Assessment test (MoCA) and Trail Making Test (TMT), but no significant differences for outcomes such as muscle strength, 5 times Sit-To-Stand test (5STS), Time Up and Go test (TUG), Single Leg Test (SLT), 30 s Chair Stand Test (30CST), and gait speed. For secondary outcomes, although the effect size was small, dance presented significant improvements in cognition (MoCA and TMT). Additionally, positive trends were observed in social engagement, quality of life (QoL), and fat-free mass. This is consistent with previous research showing that structured exercises like dance, can help delay cognitive decline (e.g. Alzheimer’s disease, and dementia) in ageing populations [21], promote social engagement [65] and QoL [10].

Four important outcomes showed statistically significant improvements (SPPB, SRT, MoCA and TMT), which aligns with wider evidence suggesting that dance-based or multimodal exercise can enhance physical function, flexibility and cognitive performance in older adults [18, 21]. For the SPPB, the estimated effects fell within the range considered clinically meaningful for community-dwelling older adults (approximately 0.5–1.0 points [66, 67]), suggesting that dance may offer potentially valuable functional benefits. However, given the variability in study quality and intervention characteristics, the clinical significance of these gains may be limited. For flexibility and cognitive outcomes (SRT, MoCA and TMT), widely accepted Minimal Clinically Important Difference thresholds are not available, making it difficult to determine whether the observed improvements translate into meaningful change. These uncertainties highlight the need for studies that establish clinically relevant benchmarks for dance-based interventions and incorporate outcomes that better capture sarcopenia-related changes.

The non-significant effect observed for 5STS in our meta-analysis contrasts with the findings of another review [68]. In our subgroup analysis, even when compared to usual care, the 12-month dance intervention did not improve performance. This may be related to the poor health status and low participation rate (51%) of the dance group at baseline assessment [53]. Another possible reason is that the dance intervention emphasized step practice rather than balance training [53], which may have limited its effectiveness in improving 5STS.

The SPPB is convenient to use and can predict sarcopenia-related outcomes [60]. Our findings indicate that dance interventions demonstrated a more benefits on SPPB compared to both walking training and usual care. This may be because dance incorporates rotations, jumps, and flexion–extension movements of different body parts, providing a more comprehensive workout [69]. In contrast, walking training typically involves repetitive lower-limb movements that engages limited muscles and joints [46]. The diverse movement patterns in dance may contribute to better coordination, balance, and overall physical function. However, variations in dance styles, intervention duration, sample characteristics, and adherence may cause heterogeneity in findings and potentially mask the potential benefits of dance interventions.

Our meta-analysis did not find beneficial effects of dance interventions on TUG, which differs from previous studies [68, 70]. It is possible that in the study by Pablo et al. [57], participants were women with sarcopenia and the control group received resistance training. Resistance training is more targeted and effective for improving outcomes in individuals with sarcopenia compared to dance. The high heterogeneity in the combined results (97%) affects the robustness of the findings, with potential sources including differences in intervention details, participant age and health status. Although we performed subgroup analyses based on control type, the limited number of studies prevented further subgroup analysis by specific exercise types, resulting in high heterogeneity. Specifically, dance significantly improved TUG compared to stretching [51], but not resistance training [57].

Although the development and implementation details of dance interventions were poorly reported, most dance interventions were designed to be accessible and easier for older adults to follow. A well-structured dance session generally consists of three phases: warm-up, main dance, and cool-down. The warm-up phase helps participants transition into the dance routine while reducing the risk of injury. The main dance phase should follow a progressive approach, with movement difficulty adjusted based on participants’ capabilities. The cool-down phase typically includes stretching exercises and deep breathing to promote relaxation and recovery. To enhance safety, continuous monitoring should be maintained throughout the entire dance activity, such as direct observation or using appropriate safety monitoring equipment. Participants should also be informed both before and during the intervention, and allowed to rest at any time if they feel tired or uncomfortable, ensuring both safety and comfort during the intervention. For implementation, most dance interventions showed good adherence and completion rates, indicating a high level of acceptance among older adults. However, home-based dance practice has not consistently provided additional benefits. Three cases of pain associated with unsupervised at-home dance training were reported [50], due to the lack of professional guidance and safety monitoring. Although such injuries may occur even under supervision, the lack of supervision can further increase risk and potentially diminish the overall benefits of dance interventions.

Future dance interventions should be well-structured, incorporate progressive difficulty adjustments, and ensure continuous safety monitoring to accommodate older adults with varying physical abilities. Additionally, it is important to focus on optimizing supervised in-class instruction, and minimizing unsupervised training as it may increase the risk of exercise-related injuries.

Older adults were generally positive about group dance interventions [38, 43]. Some participants benefited from dance interventions, such as stopping diabetes medication, healthy weight loss, and improved mobility and balance [44]. These self-reported positive outcomes support the effectiveness of dance as a health-promoting intervention. Compared to other forms of exercise, dance has unique advantages because of its connection with music. Music can make the activity more enjoyable, helping motivate older adults and evoking their emotional connections [44]. Dance also improves body control, builds confidence, and enhances self-awareness [56]. With an increasing number of older adults living alone, social isolation and loneliness have become prevalent concerns, contributing to depression and worse quality of life [65, 71]. In this context, group dance provides opportunities for social engagement and physical movement in older adults who live alone. It can reduce feelings of social isolation and loneliness, increase sense of community, and encourage social interactions and support [65].

However, dance interventions also present challenges for some older adults. For example, some may struggle with learning complex dance movements or keeping up with fast-paced rhythms, which can be frustrating [44]. Mitigation measures include the teaching style and patience of dance instructors. Instructors with good teaching skills can provide enough attention and encouragement, creating an inclusive and supportive learning environment [72]. This approach can improve the dance experience for older adults and potentially improve their adherence to the intervention. This suggests that future dance interventions should incorporate instructional design that considers individual learning styles and adaptive teaching approaches, ensuring the dance interventions meet the diverse needs and preferences of older adults [73].

Our review provides some important evidence about the contribution of group dance interventions to sarcopenia prevention and reduction. However, it cannot determine which type of dance intervention is most effective. Further research should consider variables such as cultural context, physical condition and individual preferences of older adults. Additionally, most of the studies reported only one or two of the primary outcomes, future intervention studies should include muscle mass, muscle strength, and physical performance to understand more fully the effectiveness of dance interventions in preventing and managing sarcopenia among community-dwelling older adults.

### Strengths and limitations

To our knowledge, this is the first mixed methods systematic review to examine group dance as a potential approach to prevent and manage sarcopenia in community dwelling older adults. A key strength of this review is its rigorous methodological design, including adherence to a pre-defined protocol and the application of structured quality appraisal using appropriate tools for different study designs.

A limitation is the limited number of studies included in each meta-analysis. Most pooled results were based on only 2–4 studies, evidence on the effectiveness of group dance interventions for sarcopenia therefore warrants further empirical investigation. We conducted subgroup analyses, however, the small number of studies within each subgroup may have limited their ability to explain heterogeneity or reveal meaningful patterns. Further, whilst some outcomes were statistically significant, their clinical impact may be limited. Because of the limited number of studies and heterogeneity among the included studies, we were unable to conduct a publication bias test. Several of the included studies did not clearly describe important details of the intervention, such as frequency, duration, intensity, and supervision. This limits the ability to compare interventions or interpret the potential relationship between dose and response.

Only studies published in English and Chinese were included because translation resources were not available. Our searches were limited to studies published between 2014 and 2024, which we have justified but acknowledge that this may have led to the omission of earlier but potentially relevant evidence. Positively, 2521 search results from several evidence sources provides confidence that key studies have been identified. The risk of selection bias is acknowledged, limiting generalizability of the results to the wider population, however we were keen to included contemporary evidence. Finally, for the included RCTs, we only analyzed and compared post-intervention data because follow-up outcomes were reported inconsistently across studies. Therefore, the long-term effects of dance interventions on the prevention and management of sarcopenia remain unclear.

## Conclusions and implications of review findings

Group dance interventions can significantly improve Short Physical Performance Battery (SPPB) and Sit and Reach Test (SRT) in older adults. This type of intervention also offers enjoyment and social interaction, making it safe, effective, and easily accepted by older adults. The findings of our rigorous systematic review provide valuable insights for developing sarcopenia targeted dance interventions. Effective and personalized group dance interventions are essential to support the prevention and management of sarcopenia. Future intervention studies should include muscle mass, muscle strength, and physical performance to fully understand the effectiveness of dance interventions. Further, future research should prioritise large-scale, well-designed trials with clearly defined intervention protocols, adequate follow-up, and broader inclusion criteria to better establish the clinical significance and real-world applicability of group dance interventions for sarcopenia.

## Supplementary Information

Below is the link to the electronic supplementary material.

Supplementary file1 (DOCX 1.68 MB)

## Data Availability

Data available on request from the corresponding author.
