# Supplementary material for: Group dance interventions for community dwelling older adults to prevent and treat sarcopenia: a mixed methods systematic review
Source: GeroScience. 2026 Jan 13;48(2):1701–34. doi: 10.1007/s11357-025-02072-z (PMC12972438; doi:10.1007/s11357-025-02072-z)
Supplement: Supplementary file 1 — Supplementary file1 (DOCX 1.68 MB) [file 11357_2025_2072_MOESM1_ESM.docx]

**Supplementary Material A:**

**Search Strategy**

All searches carried out 26 June 2024

**Ovid MEDLINE(R) ALL**

| NUM | SEARCH STRATEGY | RESULTS |
| --- | --- | --- |
| 1 | sarcopeni*.mp. | 20297 |
| 2 | exp muscle strength/ | 47391 |
| 3 | exp muscle, skeletal/ | 311776 |
| 4 | exp physical functional performance/ | 4532 |
| 5 | (muscle adj2 (function or loss or health or endurance or group* or wast* or progression or atrophy)).mp. | 56695 |
| 6 | (pre-sarcopenia or muscul* function or muscul* atrophy or body composition).mp. | 97721 |
| 7 | 1 or 2 or 3 or 4 or 5 or 6 | 460697 |
| 8 | exp aged/ | 3526320 |
| 9 | (older adult* or elder* or age* or senior* or geriatric* or aging* or older people or older subject or older person or senior* or "aged, 80 and over" or oldest old or centenarian* or nonagenarian* or octogenarian*).mp. | 13834480 |
| 10 | 8 or 9 | 13834480 |
| 11 | exp dancing/ | 3668 |
| 12 | (danc* or dmt or movement therapy or square stepping exercise).mp. | 15829 |
| 13 | 11 or 12 | 15829 |
| 14 | 7 and 10 and 13 | 535 |
| 15 | exp Secondary Prevention/ | 22954 |
| 16 | (prevent* or early therap* or therap*, early or risk).mp. | 5641339 |
| 17 | 15 or 16 | 5641339 |
| 18 | exp therapeutics/ | 5348016 |
| 19 | (therap* or treat* or manage*).mp. | 12082171 |
| 20 | 18 or 19 | 13887057 |
| 21 | 17 or 20 | 16364473 |
| 22 | 14 and 21 | 337 |
| 23 | limit 22 to yr="2014 -Current" | 192 |

**Embase Classic+Embase**

| NUM | SEARCH STRATEGY | RESULTS |
| --- | --- | --- |
| 1 | sarcopeni*.mp. | 32665 |
| 2 | exp muscle strength/ | 90913 |
| 3 | exp muscle mass/ | 42155 |
| 4 | exp physical performance/ | 130935 |
| 5 | (muscle adj2 (function or loss or health or endurance or group* or wast* or progression or atrophy)).mp. | 125295 |
| 6 | (pre-sarcopenia or muscul* function or muscul* atrophy or body composition).mp. | 127876 |
| 7 | 1 or 2 or 3 or 4 or 5 or 6 | 450828 |
| 8 | exp aged/ | 4057836 |
| 9 | (older adult* or elder* or age* or senior* or geriatric* or aging* or older people or older subject or older person or senior* or "aged, 80 and over" or oldest old or centenarian* or nonagenarian* or octogenarian*).mp. | 14303442 |
| 10 | 8 or 9 | 14303442 |
| 11 | exp dancing/ | 7207 |
| 12 | (danc* or dmt or movement therapy or square stepping exercise).mp. | 25830 |
| 13 | 11 or 12 | 25830 |
| 14 | 7 and 10 and 13 | 1094 |
| 15 | exp prevention/ | 1939942 |
| 16 | (prevent* or early therap* or therap*, early or risk).mp. | 7939450 |
| 17 | 15 or 16 | 8733085 |
| 18 | exp therapy/ | 11412429 |
| 19 | (therap* or treat* or manage*).mp. | 18046754 |
| 20 | 18 or 19 | 20676990 |
| 21 | 17 or 20 | 23587126 |
| 22 | 14 and 21 | 855 |
| 23 | limit 22 to yr="2014 -Current" | 517 |

**APA PsycInfo**

| NUM | SEARCH STRATEGY | RESULTS |
| --- | --- | --- |
| 1 | sarcopeni*.mp. | 531 |
| 2 | (muscle adj2 (strength or mass or performance or function or loss or health or endurance or group* or wast* or progression or atrophy)).mp. | 6679 |
| 3 | (pre-sarcopenia or muscul* function or muscul* atrophy or body composition or physical).mp. | 366184 |
| 4 | 1 or 2 or 3 | 369852 |
| 5 | exp Aging/ | 95048 |
| 6 | (older adult* or elder* or age* or senior* or geriatric* or aging* or older people or older subject or older person or senior* or "aged, 80 and over" or oldest old or centenarian* or nonagenarian* or octogenarian*).mp. | 1491655 |
| 7 | 5 or 6 | 1498967 |
| 8 | exp dance/ | 3146 |
| 9 | (danc* or dmt or movement therapy or square stepping exercise).mp. | 12253 |
| 10 | 8 or 9 | 12253 |
| 11 | 4 and 7 and 10 | 803 |
| 12 | exp prevention/ | 79075 |
| 13 | (prevent* or early therap* or therap*, early or risk).mp. | 721088 |
| 14 | 12 or 13 | 724120 |
| 15 | exp Treatment/ | 1370488 |
| 16 | (therap* or treat* or manage*).mp. | 1513572 |
| 17 | 15 or 16 | 2023386 |
| 18 | 14 or 17 | 2389110 |
| 19 | 11 and 18 | 518 |
| 20 | limit 19 to yr="2014 -Current" | 258 |

**Web of Science**

| NUM | SEARCH STRATEGY | RESULT |
| --- | --- | --- |
| 1 | TS=(sarcopeni* OR pre-sarcopenia OR muscle mass OR muscle strength OR muscle function OR muscle loss OR muscle health OR muscle endurance OR muscle group* OR muscle wast* OR muscle progression OR musculoskeletal function OR muscular atrophy OR physical performance OR body composition) | 965090  93132 |
| 2 | TS=(older adult* or elder* or age* or senior* or geriatric* or aging* or older people or older subject or older person or senior* or "aged, 80 and over" or oldest old or centenarian* or nonagenarian* or octogenarian*) | 8221430  1770571 |
| 3 | TS=(danc* or dmt or movement therapy or square stepping exercise) | 107412  50120 |
| 4 | 1 and 2 and 3 | 2292  22 |
| 5 | TS=(prevent* or early therap* or therap*, early or risk) | 6651917 |
| 6 | TS=(therap* or treat* or manage* ) | 13478703 |
| 7 | 5 or 6 | [17,346,845](https://www.webofscience.com/wos/woscc/summary/a9b2314f-63b5-490a-a24e-62f135491a2b-f700c270/relevance/1) |
| 12 | 4 and 7 | 1,823 |
|  |  | 1312 |

**CINAHL**

| NUM | SEARCH STRATEGY | RESULT |
| --- | --- | --- |
| S1 | MH sarcopenia | 4696 |
| S2 | AB ( sarcopeni* OR pre-sarcopenia OR muscle mass OR muscle strength OR muscle function OR muscle loss OR muscle health OR muscle endurance OR muscle group* OR muscle wast* OR muscle progression OR musculoskeletal function OR muscular atrophy OR physical performance ) OR TI ( sarcopeni* OR pre-sarcopenia OR muscle mass OR muscle strength OR muscle function OR muscle loss OR muscle health OR muscle endurance OR muscle group* OR muscle wast* OR muscle progression OR musculoskeletal function OR muscular atrophy OR physical performance ) | 51585 |
| S3 | S1 or S2 | 52066 |
| S4 | MH aged | 933,278 |
| S5 | AB ( older* OR elder* OR senior* OR geriatric* OR aging OR age* OR “aged, 80 and over” OR oldest OR centenarian* OR nonagenarian* OR octogenarian* ) OR TI ( older* OR elder* OR senior* OR geriatric* OR aging OR age* OR “aged, 80 and over” OR oldest OR centenarian* OR nonagenarian* OR octogenarian* ) | 1,342,072 |
| S6 | S4 or S5 | 1,861,520 |
| S7 | MH dancing | 3596 |
| S8 | AB ( danc* OR dmt OR movement therapy OR square stepping exercise ) OR TI ( danc* OR dmt OR movement therapy OR square stepping exercise ) | 8339 |
| S9 | S7 or S8 | 9441 |
| S10 | S3 and S6 and S9 | 127 |
| S11 | AB ( prevent* or early therap* or therap*, early or risk ) OR TI ( prevent* or early therap* or therap*, early or risk ) | 1,225,271 |
| S12 | MH Treatment Outcomes | 437,876 |
| S13 | MH management | 11816 |
| S14 | AB ( therap* or treat* or manage* ) OR TI ( therap* or treat* or manage* ) | 1,993,052 |
| S15 | S11 OR S12 OR S13 OR S14 | 2,904,080 |
| S16 | S10 or S15 | 78 |
| S17 | **Limiters** - Publication Date: 20140101-20241231 | 58 |

**Google scholar get 100 studies**

sarcopenia|muscle|body composition|physical

aged|aging|older|geriatric|senior|elder

dance

prevent|treatment|therapy|risk

**CNKI Get 74 studies**

**Wanfang Get 10 studies**

**Supplementary Material B:**


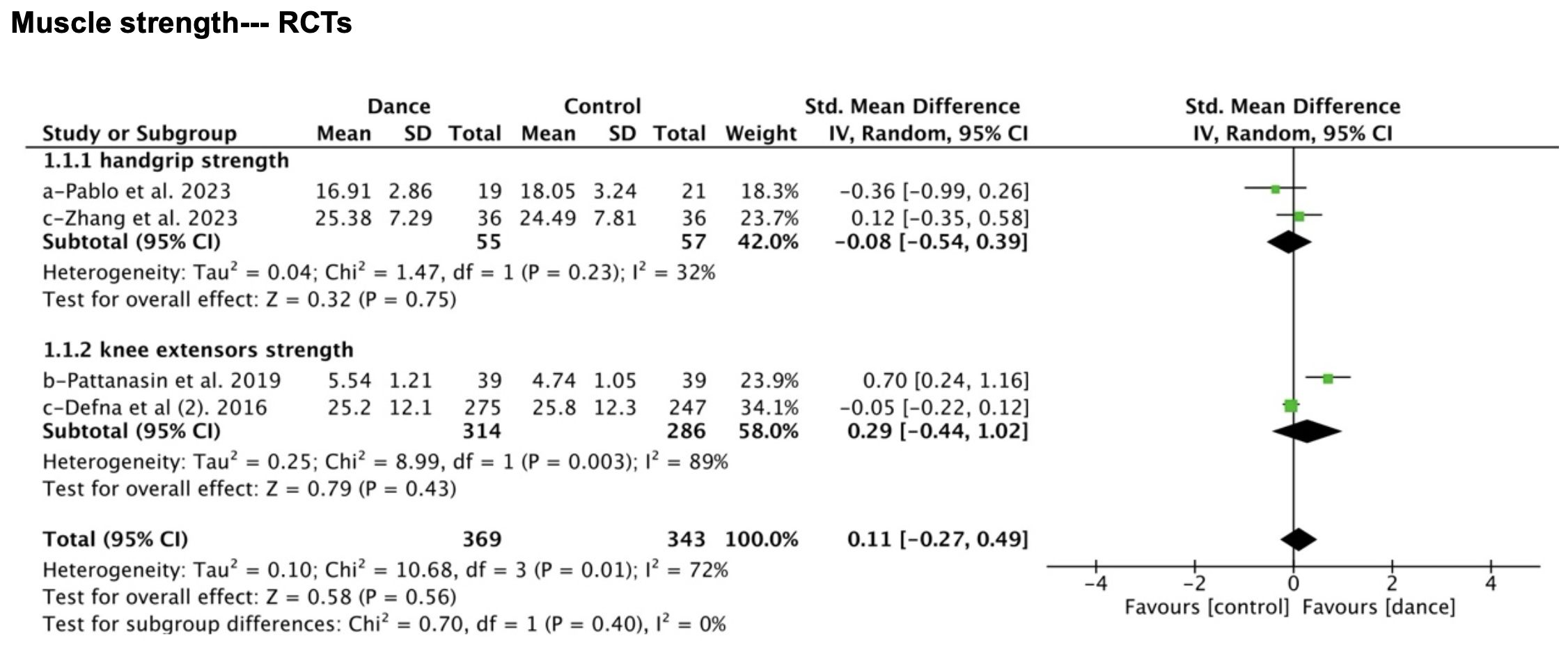


**eFigure 1. Forest plot of muscle strength** (a, exercise control; b, education control; c, usual care [routine daily activities])


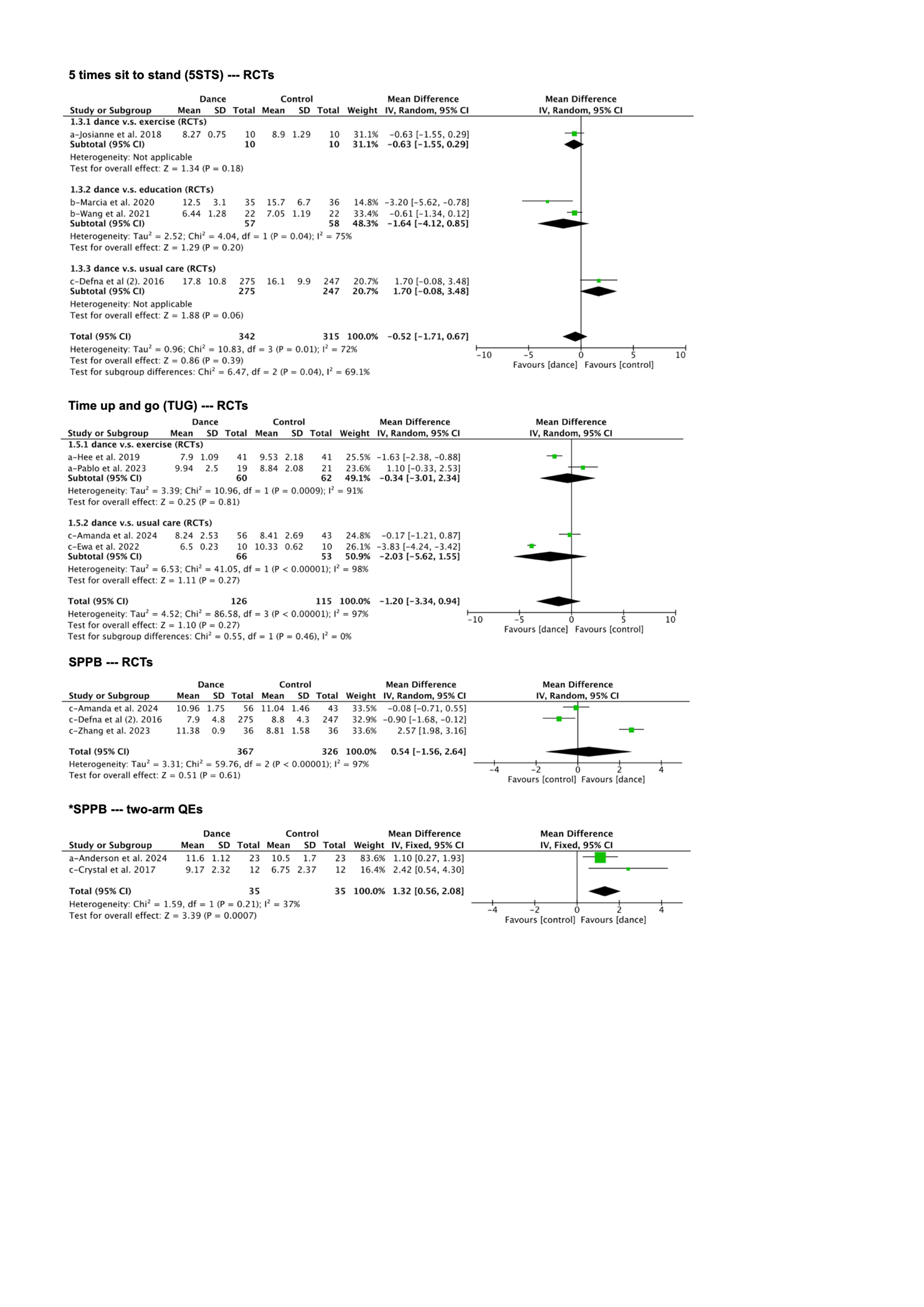

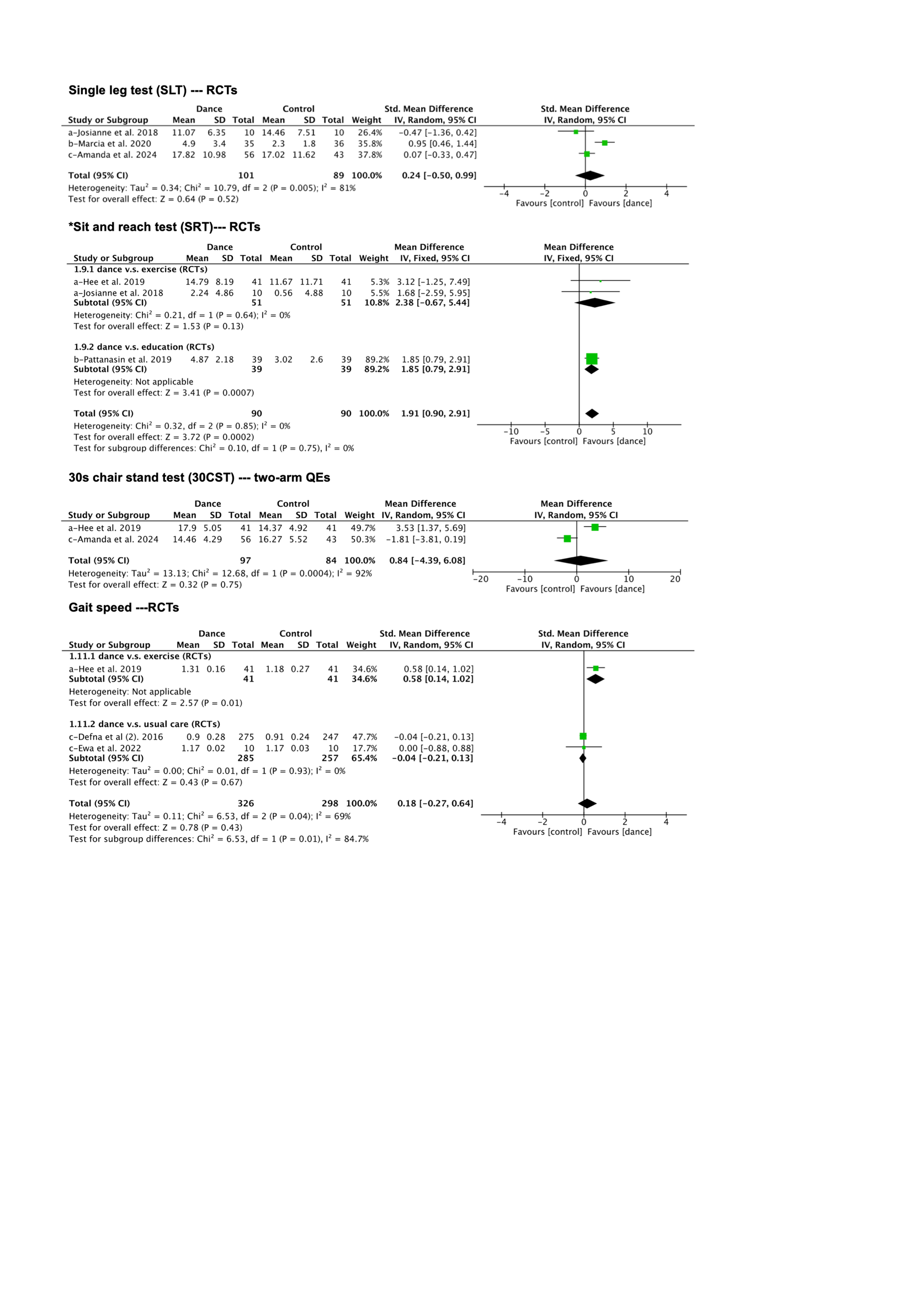


**eFigure 2. Forest plot of physical performance** (*statistically significant; a, exercise control; b, education control; c, usual care [routine daily activities].)

**Supplementary Material C:**

**eTable 1. Summary of CASP RCT assessments across domains**

| **CASP RCT Domain** | **Yes n (%)** | **No n (%)** | **Can’t tell n (%)** |
| --- | --- | --- | --- |
| D1 Research question | 13 (100) | 0 (0) | 0 (0) |
| D2 Randomization | 13 (100) | 0 (0) | 0 (0) |
| D3 Intention-to-treat analysis | 8 (61.54) | 5 (38.46) | 0 (0) |
| D4 Blind - participants | 0 (0) | 8 (61.54) | 5 (38.46) |
| D5 Blind - investigating | 7 (53.85) | 2 (15.38) | 4 (30.77) |
| D6 Blind - assess/analysis | 4 (30.77) | 0 (0) | 9 (69.23) |
| D7 Similar baseline | 13 (100) | 0 (0) | 0 (0) |
| D8 Treat equally | 13 (100) | 0 (0) | 0 (0) |
| D9 Effects reported comprehensively | 13 (100) | 0 (0) | 0 (0) |
| D10 Precision of the estimate | 7 (53.85) | 6 (46.15) | 0 (0) |
| D11 Benefits outweigh harms/costs | 13 (100) | 0 (0) | 0 (0) |
| D12 Results be applied | 13 (100) | 0 (0) | 0 (0) |
| D13 Great value than interventions | 0 (0) | 0 (0) | 13 (100) |

**eTable 2. Summary of ROBINS-I assessments across domains**

| **ROBINS-I Domain** | **Low n (%)** | **Moderate n (%)** | **Serious/Critical n (%)** |
| --- | --- | --- | --- |
| D1 Confounding | 0 (0) | 4 (57.14) | 3 (42.86) |
| D2 Selection of participants | 5 (71.43) | 2 (28.57) | 0 (0) |
| D3 Classification of interventions | 5 (71.43) | 2 (28.57) | 0 (0) |
| D4 Deviations from intended interventions | 2 (28.57) | 5 (71.43) | 0 (0) |
| D5 Missing data | 2 (28.57) | 5 (71.43) | 0 (0) |
| D6 Measurement of outcomes | 0 (0) | 6 (85.71) | 1 (14.29) |
| D7 Selection of reported result | 4 (57.14) | 3 (42.86) | 0 (0) |

**eTable 3. Summary of CASP Qualitative assessments across domains**

| **CASP Qual Domain** | **Yes n (%)** | **No n (%)** | **Can't tell n (%)** |
| --- | --- | --- | --- |
| D1 Clear aims | 4 (100) | 0 (0) | 0 (0) |
| D2 Appropriate methodology | 4 (100) | 0 (0) | 0 (0) |
| D3 Appropriate design | 4 (100) | 0 (0) | 0 (0) |
| D4 Appropriate recruitment strategy | 3 (75) | 0 (0) | 1 (25) |
| D5 Data collection | 4 (100) | 0 (0) | 0 (0) |
| D6 Relationship between researcher and participants | 1 (25) | 0 (0) | 3 (75) |
| D7 Ethical issues | 4 (100) | 0 (0) | 0 (0) |
| D8 Rigorous data analysis | 3 (75) | 0 (0) | 1 (25) |
| D9 Clear statement of findings | 4 (100) | 0 (0) | 0 (0) |
| D10 Research value | 4 (100) | 0 (0) | 0 (0) |

**Supplementary Material D:**

**PRISMA 2020 Checklist.**
This table provides a completed PRISMA 2020 checklist, indicating the page or section in the manuscript where each reporting item is addressed.

| **Section and Topic** | **Item #** | **Checklist item** | **Location where item is reported** |
| --- | --- | --- | --- |
| **TITLE** | | |  |
| Title | 1 | Identify the report as a systematic review. | 1 |
| **ABSTRACT** | | |  |
| Abstract | 2 | See the PRISMA 2020 for Abstracts checklist. | 1 |
| **INTRODUCTION** | | |  |
| Rationale | 3 | Describe the rationale for the review in the context of existing knowledge. | 2 |
| Objectives | 4 | Provide an explicit statement of the objective(s) or question(s) the review addresses. | 2 |
| **METHODS** | | |  |
| Eligibility criteria | 5 | Specify the inclusion and exclusion criteria for the review and how studies were grouped for the syntheses. | 3 |
| Information sources | 6 | Specify all databases, registers, websites, organisations, reference lists and other sources searched or consulted to identify studies. Specify the date when each source was last searched or consulted. | 3 |
| Search strategy | 7 | Present the full search strategies for all databases, registers and websites, including any filters and limits used. | 3, Supplementary Material |
| Selection process | 8 | Specify the methods used to decide whether a study met the inclusion criteria of the review, including how many reviewers screened each record and each report retrieved, whether they worked independently, and if applicable, details of automation tools used in the process. | 4 |
| Data collection process | 9 | Specify the methods used to collect data from reports, including how many reviewers collected data from each report, whether they worked independently, any processes for obtaining or confirming data from study investigators, and if applicable, details of automation tools used in the process. | 4 |
| Data items | 10a | List and define all outcomes for which data were sought. Specify whether all results that were compatible with each outcome domain in each study were sought (e.g. for all measures, time points, analyses), and if not, the methods used to decide which results to collect. | 4 |
|  | 10b | List and define all other variables for which data were sought (e.g. participant and intervention characteristics, funding sources). Describe any assumptions made about any missing or unclear information. | 4 |
| Study risk of bias assessment | 11 | Specify the methods used to assess risk of bias in the included studies, including details of the tool(s) used, how many reviewers assessed each study and whether they worked independently, and if applicable, details of automation tools used in the process. | 4 |
| Effect measures | 12 | Specify for each outcome the effect measure(s) (e.g. risk ratio, mean difference) used in the synthesis or presentation of results. | 4-5 |
| Synthesis methods | 13a | Describe the processes used to decide which studies were eligible for each synthesis (e.g. tabulating the study intervention characteristics and comparing against the planned groups for each synthesis (item #5)). | 4-5 |
|  | 13b | Describe any methods required to prepare the data for presentation or synthesis, such as handling of missing summary statistics, or data conversions. | 4-5 |
|  | 13c | Describe any methods used to tabulate or visually display results of individual studies and syntheses. | 4-5 |
|  | 13d | Describe any methods used to synthesize results and provide a rationale for the choice(s). If meta-analysis was performed, describe the model(s), method(s) to identify the presence and extent of statistical heterogeneity, and software package(s) used. | 4-5 |
|  | 13e | Describe any methods used to explore possible causes of heterogeneity among study results (e.g. subgroup analysis, meta-regression). | 4-5 |
|  | 13f | Describe any sensitivity analyses conducted to assess robustness of the synthesized results. | 4-5 |
| Reporting bias assessment | 14 | Describe any methods used to assess risk of bias due to missing results in a synthesis (arising from reporting biases). | 4 |
| Certainty assessment | 15 | Describe any methods used to assess certainty (or confidence) in the body of evidence for an outcome. | 4 |
| **RESULTS** | | |  |
| Study selection | 16a | Describe the results of the search and selection process, from the number of records identified in the search to the number of studies included in the review, ideally using a flow diagram. | Figure 1 |
|  | 16b | Cite studies that might appear to meet the inclusion criteria, but which were excluded, and explain why they were excluded. | NA |
| Study characteristics | 17 | Cite each included study and present its characteristics. | 5-10, Table 1 |
| Risk of bias in studies | 18 | Present assessments of risk of bias for each included study. | 11, Figure 4 |
| Results of individual studies | 19 | For all outcomes, present, for each study: (a) summary statistics for each group (where appropriate) and (b) an effect estimate and its precision (e.g. confidence/credible interval), ideally using structured tables or plots. | Table 1 |
| Results of syntheses | 20a | For each synthesis, briefly summarise the characteristics and risk of bias among contributing studies. | 7-10 |
|  | 20b | Present results of all statistical syntheses conducted. If meta-analysis was done, present for each the summary estimate and its precision (e.g. confidence/credible interval) and measures of statistical heterogeneity. If comparing groups, describe the direction of the effect. | 7-10 |
|  | 20c | Present results of all investigations of possible causes of heterogeneity among study results. | 7-10 |
|  | 20d | Present results of all sensitivity analyses conducted to assess the robustness of the synthesized results. | 7-10 |
| Reporting biases | 21 | Present assessments of risk of bias due to missing results (arising from reporting biases) for each synthesis assessed. | 7-11 |
| Certainty of evidence | 22 | Present assessments of certainty (or confidence) in the body of evidence for each outcome assessed. | 7-10 |
| **DISCUSSION** | | |  |
| Discussion | 23a | Provide a general interpretation of the results in the context of other evidence. | 12,13 |
|  | 23b | Discuss any limitations of the evidence included in the review. | 14 |
|  | 23c | Discuss any limitations of the review processes used. | 14 |
|  | 23d | Discuss implications of the results for practice, policy, and future research. | 14,15 |
| **OTHER INFORMATION** | | |  |
| Registration and protocol | 24a | Provide registration information for the review, including register name and registration number, or state that the review was not registered. | 3 |
|  | 24b | Indicate where the review protocol can be accessed, or state that a protocol was not prepared. | 3 |
|  | 24c | Describe and explain any amendments to information provided at registration or in the protocol. | 3 |
| Support | 25 | Describe sources of financial or non-financial support for the review, and the role of the funders or sponsors in the review. | Title page |
| Competing interests | 26 | Declare any competing interests of review authors. | Title page |
| Availability of data, code and other materials | 27 | Report which of the following are publicly available and where they can be found: template data collection forms; data extracted from included studies; data used for all analyses; analytic code; any other materials used in the review. | Title page |

*From:*  Page MJ, McKenzie JE, Bossuyt PM, Boutron I, Hoffmann TC, Mulrow CD, et al. The PRISMA 2020 statement: an updated guideline for reporting systematic reviews. BMJ 2021;372:n71. doi: 10.1136/bmj.n71

For more information, visit: <http://www.prisma-statement.org/>
